# QTL analysis identified two major all-internodes solidness loci from a completely solid-stemmed spring wheat line

**DOI:** 10.3389/fpls.2022.1035620

**Published:** 2022-11-15

**Authors:** Raman Dhariwal, Colin W. Hiebert, Harpinder S. Randhawa

**Affiliations:** ^1^ Agriculture and Agri-Food Canada, Lethbridge Research and Development Centre, Lethbridge, AB, Canada; ^2^ Agriculture and Agri-Food Canada, Morden Research and Development Centre, Morden, MB, Canada

**Keywords:** wheat stem sawfly, solid stem wheat, pith development, SNP mapping, linkage map, QTL mapping

## Abstract

The culms of solid-stemmed wheat cultivars are filled with “pith” – a parenchymatous tissue largely composed of soft, spongy, and compact parenchyma cells. Breeding solid-stemmed cultivars is the most effective way to decrease the detrimental impact of wheat stem sawfly (WSS), *Cephus cinctus* Norton (Hymenoptera: Cephidae) on wheat production. Although a major solid stem gene has been previously identified from durum wheat, it produces an intermediate level of stem solidness in common wheat which is insufficient to provide the required level of WSS resistance. The maximum resistance is achieved when stems are totally filled with pith. Thus, to identify a secondary source of solidness in common wheat, we developed three mapping populations from wheat cvs. Sadash, ‘AAC Innova’ and ‘AAC Cameron’, each crossed separately with P2711, a completely solid-stemmed hexaploid wheat breeding line. All populations were genotyped using either wheat 15K or 90K Infinium iSelect SNP Assay and high-density linkage maps were generated from individual populations along with consensus maps for chromosomes 3B and 3D from all populations. ‘Sadash/P2711’ and ‘AAC Innova/P2711’ populations were subjected to extensive phenotyping in ≥3 environments followed by quantitative trait loci (QTL) analyses using population-specific and consensus linkage maps. We identified two major solid stem QTLs in the distal regions of chromosome arms 3BL and 3DL in both populations in addition to several population-specific or common minor QTLs. Internode-specific QTL analyses detected both major QTLs of chromosomes 3B and 3D across internodes, from top to bottom of the stalk, but minor QTLs were largely detected in upper or middle internodes. Our results suggest that both major QTLs are sufficient to develop highly solid-stemmed cvs; however, the minor loci, which additively enhance the pith expression, can be coupled with major genes to achieve a complete solid stem phenotype in common wheat. Comparative and haplotype analyses showed that the 3B locus is homoeologous to 3D, the former being mapped to a 1.1 Mb genomic region. Major QTLs identified in this study can be incorporated in modern wheat cultivars to achieve maximum WSS resistance from high pith expression.

## Introduction

Wheat stem sawfly (WSS), *Cephus cinctus* Norton (Hymenoptera: Cephidae), imposes severe economic barriers to wheat (*Triticum aestivum* L.) production in the northern great plains of North America and many other wheat-producing regions around the globe (Morrill et al., 1992; Talbert et al., 2014). WSS females deposit eggs in hollow stems soon after emergence from the infested stubble of the previous year’s crop following mating, usually in the early crop growing season (Beres et al., 2011a; Talbert et al., 2014; Subedi et al., 2020). These eggs hatch within 5–7 d of deposition and the resulting larvae begins feeding upon the vascular bundle tissues within the stalk (Ainslie, 1929). Larvae feed on the vascular bundles, make tunnel across internodes in the stem, interfere with the stem storage and transfer of water and nutrients to the developing grains, and reduce photosynthesis and grain yield (Macedo et al., 2007). In recent years, WSS has not only expanded its infestation area outside its traditional territories (Lesieur et al., 2016) but has also become a constant economically challenging insect pest in North America (Subedi et al., 2020). WSS-infested plants showed 5–30% kernel weight reductions in field and greenhouse conditions (Wallace et al., 1973; Delaney et al., 2010). In high WSS pressure, up to ~15% additional yield loss can be observed when infested/cut stems are lodged on the ground or healthy stems in field conditions, reaching a total loss of around 30–35% (Beres et al., 2007; Bekkerman and Weaver, 2018). Harvest losses attributed to WSS are estimated to exceed $350 million annually in North America alone (Beres et al., 2011b).

Due to the specific lifecycle and presence of alternate hosts, WSS control measures are limited (Weiss and Morrill, 1992; Beres et al., 2011a; Talbert et al., 2014). Adult females emerge for a short period, and WSS larvae feed and develop inside the stem where they are protected from the external environment including insecticide applications, thus pest management strategies primarily rely on host plant resistance (Talbert et al., 2014; Biyiklioglu et al., 2018). Initial research on *Cephus pygmaeus* (L.), a species of the European WSS, in the 1920s revealed less damage in wheat plants having stems filled with pith (Schegolev, 1926; Varella et al., 2017). These wheat cultivars are referred to as solid stemmed wheat and exhibit resistance by deterring oviposition for WSS adult females, impeding larval migration and growth or death of larvae inside stems, which greatly reduce stem cutting and WSS population abundance (Wallace et al., 1973). The use of solid-stemmed cultivars is considered the most effective way to minimize WSS damage (Beres et al., 2013).

Research on stem solidness has been the major focus of WSS resistance in breeding programs for over several decades. The first genetic study on solid stem inheritance in wheat was published in 1905 by Biffen (1905) followed by a series of classical genetics studies on different species and cultivars in succeeding years (Engledow, 1920; Engledow, 1923; Engledow and Hutchinson, 1925; Thompson, 1931; Kemp, 1934; Goytia Y Angulo, 1935; Yamashita, 1937; Platt, 1941; Putnam, 1942; Platt and Larson, 1944; Larson, 1952; Mc Neal, 1956; Mcneal et al., 1957; Larson, 1959a; Larson, 1959b; Larson, 1959c; Larson and Macdonald, 1959a; Larson and Macdonald, 1959b; Mc Neal, 1961; Bozzini and Avanzi, 1962; Larson and Macdonald, 1962; Mckenzie, 1965). These studies not only helped in the identification of genomes and chromosomes carrying genetic factors/genes and their number variation that determines stem solidness or hollowness but also provided a solid background work on resistance to the WSS due to solid stem. These studies also demonstrated that stem solidness was most common in the tetraploid species, while unknown in diploids (Vavilov, 1951) and rare in hexaploids (Platt et al., 1941). Moreover, durum wheat was found more solid than common wheat (Kemp, 1934). This steered intensive efforts for introgression or transfer of the complete stem solidness from tetraploid to hexaploid species as a resistance source to control the WSS, which resulted in large failures initially (Platt and Larson, 1944; Larson, 1959c) but succeeded later on in the specific genetic background (Mc Neal, 1961). Instantaneously, wheat breeding for resistance to the WSS had become synonymous with breeding for stem solidness (Talbert et al., 2014). The first North American hexaploid (hard red spring) wheat cultivar released from these efforts in Canada was ‘Rescue’, which was quickly picked up by the growers and grown throughout the wheat stem sawfly impacted areas in the late 1940s and early 1950s (Talbert et al., 2014). In the following decades, superior solid-stemmed cultivars, particularly durum wheat and some common wheat, quickly followed and dominated acreage in WSS-infested regions in North America (Talbert et al., 2014).

Genetic experiments started in the early 21^st^ century in hexaploid wheat have identified a major stem solidness QTL *Qss.msub-3BL* (Cook et al., 2004) and a moderate effect locus *Qss.msub-3DL* (Lanning et al., 2006). The 3BL major locus has also been detected in durum wheat (*T. turgidum* var. durum), which was later designated as *Solid-stem locus 1* (*SSt1*) (Houshmand et al., 2007). Recently, Cook et al. (2017) revealed several new haplotypes at *Qss.msub.3BL* such as one for the early stem solidness. In addition to 3BL and 3DL loci, a number of minor QTLs that affects solid stem expression have been detected in common and durum wheat (Larson and Macdonald, 1959a; Sherman et al., 2010; Varella et al., 2015; Nilsen et al., 2017; Varella et al., 2019). Kebrom et al. (2012) discovered that a ‘*tiller inhibition*’ (*tin*) gene (Atsmon and Jacobs, 1977) that is tightly linked with the simple sequence repeat (SSR) marker *gwm136* on chromosome arm 1AS (Spielmeyer and Richards, 2004) is associated with precocious internode development and solidness in basal internodes of wheat plants. Later in a separate study, Hyles et al. (2017) found that *tin* encodes a cellulose synthase-like (Csl) protein which increases lignification associated with stronger stems in *tin* wheat plants. In 2017, Oiestad et al. (2017) reported that stem solidness at *Qss.msub-3BL* locus is associated with gene expression changes related to lignin biosynthesis. Recently, Nilsen et al. (2020) identified *TdDof*, which encodes Dof zinc finger protein, as the causal gene that controls stem solidness at the *SSt1* locus on chromosome 3B in durum. They showed that copy number gain of *TdDof* correlates with its increased expression and the solid-stem phenotype.

Despite the progress made in the research, breeding hexaploid wheat for stem-solidness is a challenge due to inconsistent expression of pith in hexaploid genetic backgrounds (Cook et al., 2004; Lanning et al., 2006). Stems of genotypes containing the allele for solidness often are of an intermediate solidness in common wheat that does not confer sufficient resistance to the WSS (Wallace et al., 1973; Morrill et al., 1992; Lanning et al., 2006). This is perhaps because most solid‐stemmed common wheat genotypes carry a single solid stem gene from the Portuguese landrace ‘S‐615’ (Varella et al., 2019) which does not fully express in the hexaploid background due to some inhibitors and epistatic interactions. Maximum resistance from stem solidness can be obtained when the stems are completely filled with pith (Holmes, 1977). Thus, to develop complete solid-stemmed cultivars of hexaploid wheat, it is necessary to identify the secondary sources of stem solidness.

P2711 is a completely solid-stemmed (**Supplementary Video S1**) hexaploid spring wheat breeding line from South Africa but has not yet been characterized for stem solidness. Thus, the identification of additional gene(s) that increase the stem-solidness in P2711 and their linked markers would be useful for common wheat breeding. For this study, we developed two doubled haploid (DH) populations and one recombinant inbred line (RIL) population from Canadian common wheat cvs. Sadash (Sadasivaiah et al., 2009), ‘AAC Innova’ (Randhawa et al., 2015) and ‘AAC Cameron’ (Fox et al., 2016), each crossed with P2711 (Farzand et al., 2022). The objectives of this study were to identify secondary QTLs and their markers for stem-solidness from P2711 that would enhance the effect of the primary QTL and compare identified QTLs with the ones reported previously.

## Materials and methods

### Plant material

Three spring wheat mapping populations, two DH and one RIL, were developed by crossing Canadian common wheat cvs. Sadash, ‘AAC Innova’ and ‘AAC Cameron’, each used as female with P2711 as a male parent. F_1s_ produced from ‘Sadash/P2711’ and ‘AAC Innova/P2711’ crosses were used for doubled haploid production using the wheat-maize pollination technique (Suenaga and Nakajima, 1989) at the Agriculture and Agri-Food Canada (AAFC), Lethbridge Research and Development Centre (LeRDC), Lethbridge, AB, Canada. F_1s_ produced from the ‘AAC Cameron/P2711’ cross were used to produce the RIL population as described earlier (Farzand et al., 2022). The numbers of recombinant lines produced from these three crosses were 180 (‘Sadash/P2711’), 374 (‘AAC Innova/P2711’), and 252 (‘AAC Cameron/P2711’).

Sadash is a hollow-stemmed, white-grained, semi-dwarf, high-yielding, soft white spring wheat cultivar which belongs to the ‘Canada Western Soft White Spring’ (CWSWS) market class. It was developed using the traditional plant breeding method from the cross ‘SWS207/SWS208//SWS214’ made at the AAFC-LeRDC in 1997. ‘AAC Innova’ is a hollow-stemmed, white-grained, semi-dwarf, soft white spring type cultivar which belongs to the ‘Canada Western Special Purpose’ (CWSP) market class. ‘AAC Innova’ originated from the cross ‘AC Andrew/N9195’ made at AAFC-LeRDC in 2001 and was developed using a modified bulk breeding technique (Randhawa et al., 2015). ‘AAC Cameron’ is a hollow-stemmed, red-grained, tall and high-yielding spring wheat cultivar with good agronomic, disease, and end-use quality characteristics which belongs to the ‘Canada Western Red Spring’ (CWRS) market class (Fox et al., 2016). ‘AAC Cameron’ was developed using the modified pedigree breeding method from the complex cross ‘D1125/Alsen//BW346/3/BW370/99B60-EJ26’ made at AAFC, Cereal Research Centre (CRC), Winnipeg, Manitoba (MB) in 2004 (Fox et al., 2016). Male parent P2711 is a completely solid-stemmed (for all internodes), semi-dwarf, red-grained hexaploid spring wheat breeding line from South Africa but has not yet been characterized for stem solidness.

Common wheat cv Lillian (Depauw et al., 2005) and durum cv ‘Golden Ball’ (Clark et al., 1922) were used as checks for comparisons. Lillian is a hard red spring semi-solid-stemmed wheat cultivar which exhibits reduced WSS cutting (Depauw et al., 2005), while Golden Ball is a completely solid-stemmed durum cultivar (Kemp, 1934).

Seeds of cultivars used as checks and parents of the mapping populations were accessed from the Wheat Breeding core collection at AAFC-LeRDC. Seeds of parents, DHs and RILs produced in this study are preserved at AAFC-LeRDC and available upon request. All other cultivars used in this study are preserved at the Plant Gene Resources of Canada (PGRC) seed genebank based at AAFC’s Saskatoon Research and Development Centre, Saskatchewan, Canada.

### Trial environments and field experiments

‘Sadash/P2711’ recombinant doubled haploid lines, their parents and check cultivars were grown in three environments at AAFC-LeRDC, Lethbridge (49°41′N, 112°49′W) in Alberta, Canada. The three environments included: (i) field trial in 2014 (LTFD14), (ii) field trial in 2019 (LTFD19), and (iii) greenhouse trial in 2019 (LTGH19). ‘AAC Innova/P2711’ recombinant doubled haploid lines, their parents and check cultivars were grown in one and three environments, respectively, in Lincoln (43°63′S, 172°46′E), New Zealand and AAFC-LeRDC, Lethbridge, AB. The four environments included: (i) Lincoln, New Zealand field trial in 2013 (NZFD13), (ii) Lethbridge field trial in 2015 (LTFD15), (iii) Lethbridge greenhouse trial in 2015 (LTGH15), and (iv) Lethbridge field trial in 2019 (LTFD19). ‘AAC Cameron/P2711’ RILs, their parents and check cultivars were grown in the field in one and two environments, respectively, in Lincoln, New Zealand and AAFC-LeRDC, Lethbridge, AB. The three environments included: (i) F_4_ field trial in Lethbridge in 2015 (LTFD15), (ii) F_5_ field trial in Lincoln, New Zealand in 2016 (NZFD16), and (iii) F_6_ field trial at AAFC-Lethbridge in 2017 (LTFD17). Field trials in both locations were conducted on conventionally managed fields, while greenhouse experiments were conducted using a 16/8h light/dark photoperiod and the day/night temperature of 23/18°C. Single 3-m-long rows of each line/genotype were seeded in late May in a randomized block design in all Lethbridge environments. Seeding density was ~250 seeds per row with 23.5 cm row spacing. Hill plots were seeded in mid-October in New Zealand, while two pots were seeded, each with two plants, for each line/genotype in greenhouse experiments.

### Stem solidness assessment

Solid stem assessment in all trial experiments, except in the New Zealand field trial, was done following (Depauw and Read, 1982) and a modified phenotyping method (Dhariwal et al., 2017). Briefly, three to five plants from the center of each plot/row were pulled from the field at maturity and moved to the greenhouse where they were left on open benches for a few weeks for complete drying before rating for stem-solidness/pith expression. Five tillers, separately from each of the three to five plants (at least 5 x 3 = 15 total tillers or stems), were dissected longitudinally and four upper internodes (in top-down order: I1: internode 1/peduncle, I2: internode 2, I3: internode 3, and I4: internode 4/lowest internode scored) were rated for a stem-solidness (S) score of 1–5 (1 for hollow stem – no pith development, 2 for some degree of pith development, 3 for the large hollow tunnel in the stem or a huge cavity at a particular point, 4 for the size of hollow tunnel equivalent to a pencil lead, and 5 for completely solid stem). Scores of each internode across all tillers/stems were averaged to obtain a rating score per internode. While stem solidness score from each internode was utilized separately for QTL analysis to identify internode-specific loci, a plot/line score obtained by averaging all four internode scores was utilized for combined QTL analysis for stem solidness. Similarly, the rating was done for greenhouse-grown plants but the number of plants and tillers/stems per line varied. At least one plant and 5 tillers or stems were rated in the greenhouse.

In New Zealand field trials, solid-stem rating was done in the field by cutting erect wheat plants/stems at several points using sharp scissors. A subjective rating was given to lines on visual bases. Using this method, the best solid-stemmed line was rated with a solid stem score of 3 while the worst with a solid stem score of 1.

### Phenotypic data analysis

The analysis of variance (ANOVA) of solid stem for each environment and pooled data was assessed using the line solid stem score as demonstrated earlier by Basnet et al. (2012) and (Farzand et al., 2022). Briefly, ANOVA was conducted by treating genotypes within environments as random effect factors, while experiments as fixed effect factors using the packages ‘lme4’ (version 1.1.27.1) (Bates et al., 2015) and ‘lattice’ (version 0.20-45) (Sarkar, 2008) in R (version 4.0.3) (R Core Team, 2013). Covariance parameter estimates as unbiased genetic variance component estimates were used for calculating broad sense heritability (*H^2^*) as the ratio of genetic variance to phenotypic variance following Knapp et al. (1985). Pearson correlation plots, histograms with normal curves and scatterplots were estimated and plotted using the R packages GGally (Schloerke et al., 2018), randomForest (Liaw and Wiener, 2002) and ggplot2 (Wickham, 2016) in R (version 4.0.3) (R Core Team, 2013). Least-square means (LS means) for stem-solidness score (internodes and line) were estimated for each recombinant doubled haploid line using Microsoft’s Excel program.

### Genotyping, linkage mapping and quantitative trait loci analysis

For isolation of DNA from leaf samples, four seeds were sown from each genotype in 96 cell seed planting trays (parents and checks repeated over all trays) in a soil mixture of Turface (9.07 kg), Peat Moss (0.907 kg) and Vermiculite (0.06 m3). Leaf tissue samples were collected from 10 days old seedlings followed by isolation of DNA using DNeasy 96 Plant Kit (Qiagen Inc., Valencia, CA, USA) following Dhariwal et al. (2018). Quant-iT™ PicoGreen^®^ dsDNA Assay Kit (Thermo Fisher Scientific Inc., Bartlesville, OK, USA) was used to quantify DNA samples followed by dilutions of DNA samples to 50 ng/µl.

‘Sadash/P2711’ population, its parents and checks were genotyped using Wheat 15K Illumina Infinium SNP array from TraitGenetics GmbH, Germany. ‘AAC Innova/P2711’ and ‘AAC Cameron/P2711’ populations, their parents and checks were genotyped using a 90K Illumina Infinium array (Wang et al., 2014). Single nucleotide polymorphism (SNP) genotyping data were analyzed using Genotyping module of GenomeStudio software (Illumina Inc., CA, USA) as described previously (Dhariwal et al., 2020). Briefly, high quality SNPs were selected from the list of all SNPs evaluated for genotyping using following filters in GenomeStudio (i) Call Freq: >0.50, (ii) Minor Allele Freq: >0.03, (iii) AA Freq:!=1, (iv) AB Freq:!=1, and (v) BB Freq:!=1. Monomorphic and SNPs that deviated significantly from the 1:1 ratio were removed by comparing the parental profile and using the χ2 test, respectively.

‘Sadash/P2711’ population genotyping data was transformed into the mapping data format “ABH” (A = Sadash, B = P2711, H = heterozygous). Subsequently, the JoinMap^®^ 4.0 program (Van Ooijen, 2006) was used for pre-mapping of the SNPs with respect to the verification of segregation patterns, the initial formation of linkage groups (LGs) and the preliminary position of the markers on the chromosomes using the default grouping settings and the mapping algorithm ML (maximum likelihood). The final map position of the markers and the genetic distances between the markers were then optimized manually regarding the number of crossovers (as low as possible) and the length of the linkage group (as short as possible) using the ABH mapping data file in Excel and MapManager QTX (parameters: linkage evaluation - double haploid, search linkage criterion - *p*=0.05, map function - Kosambi, cross-type - line cross) (Manly et al., 2001). Some markers were eliminated from the final mapping if they spread the linkage group due to too many crossovers.

Markers belonging to the ‘AAC Innova/P2711’ population were assigned to linkage groups (LGs) using MSTmap (version 2.0) software (Wu et al., 2008) at default grouping settings and mapping function Kosambi (Kosambi, 1943) following Dhariwal and Randhawa (2022) and Farzand et al. (2021). Wheat chromosomes to LGs were assigned using the wheat 90K consensus SNP map (Wen et al., 2017). Double recombinants were corrected after re-scoring genotyping data manually in Microsoft Excel followed by recalculating the linkage map using MapDisto software (version 1.7.7.011) (Lorieux, 2012). Two or more linkage groups generated for the same chromosome were tried to merge into a single linkage group using less stringent cut-off values (r max > 0.3) using MapDisto. Linkage mapping of the ‘AAC Cameron/P2711’ population was done as described earlier (Farzand et al., 2022). Four consensus maps were generated for each of the chromosomes 3B and 3D separately utilizing individual LG maps of three populations using the R package LPmerge (version 1.7) (Endelman and Plomion, 2014). Final consensus maps for chromosomes 3B and 3D were selected based on the lowest mean and standard deviation for root-mean-squared error (RMSE) between the consensus map and the linkage maps. Whole genome linkage map plots illustrating SNP density were drawn using the R package LinkageMapView (version 2.1.2) (Ouellette et al., 2018).

Composite interval mapping (CIM) to identify the main effect solid stem QTLs (for the combined effect of all internodes or whole stem as well as internode specific analyses) in all individual environments and pooled (across environments) data collected for each population were conducted separately following Dhariwal and Randhawa (2022). The regression method “forwards and backwards cofactor (*p* < 0.05)” was used for CIM in the software package QTL Cartographer (version 1.6) (Zeng, 1993; Zeng, 1994) following Nilsen et al. (2017). Significant QTLs were declared using QTL thresholds estimated using 1000 permutations at a significance level of *p* = 0.05. QTL confidence intervals were determined using 1-LOD and 2-LOD support intervals as 95% and 99% CI (Lander and Botstein, 1989). Linkage bins possessing two or more QTLs within 10.0 cM were treated as a single QTL region (Dhariwal et al., 2020). Consecutively chromosome 3B and 3D consensus maps and solid stem LS mean obtained for each genotype were also used for QTL analysis using QTL Cartographer (version 1.6). Two-locus QTL analysis was carried out to map epistatic QTLs using QTLNetwork (version 2.0) (Yang et al., 2008) at default parameters. Linkage maps illustrating homologous relationships across populations/consensus map, QTL positions and LOD contour across environments for line solid stem score (S) and internode score (I) were drawn using MapChart software (version 2.32) (Voorrips, 2002).

### Assessment of syntenic relationship, assignment of QTL genomic intervals and mapping of candidate gene

Syntenic relationships of 3B and 3D linkage maps of ‘Sadash/P2711’ and ‘AAC Innova/P2711’ populations with physical maps of chromosomes 3B and 3D (reference genome assembly PGSBv2.1) of Canadian solid-stemmed spring wheat cultivar ‘CDC Landmark’ (pedigree: Unity/Waskada//Alsen/Superb) was assessed using BLAST search following Dhariwal et al. (2021) and Dhariwal and Randhawa (2022). Briefly, chromosome 3B (GenBank accession: LR877316.1) and 3D (GenBank accession: LR877317.1) reference sequences of cv ‘CDC Landmark’ were downloaded from the National Center for Biotechnology Information (NCBI), which were BLAST searched (with at least 99% identity and 100% query coverage) utilizing probe sequences of SNP markers [wheat 90K iSelect SNP array markers (Wang et al., 2014) and markers unique to 15K Wheat Infinium array (Soleimani et al., 2020)], which were mapped to 3B and 3D linkage groups of ‘Sadash/P2711’ and/or ‘AAC Innova/P2711’ populations, using NCBI standalone BLAST program (Altschul et al., 1990). Genomic coordinates identified from BLAST results were used to decipher syntenic relationships of 3B and 3D linkage maps of ‘Sadash/P2711’ and ‘AAC Innova/P2711’ populations with ‘CDC Landmark’ genome using Windows software program Strudel (version 1.15.08.25) (https://ics.hutton.ac.uk/strudel/).

Similar to the BLAST search for syntenic relationships, probe sequences of all the SNPs (wheat 90K iSelect SNP array and markers unique to 15K Wheat Infinium array) that were mapped into QTL intervals in this study were also BLAST searched against the Chinese Spring wheat reference genome sequence (IWGSC RefSeq v2.1) to identify the QTL genomic intervals on wheat chromosomes. cDNA sequence of durum solid stem gene *TdDof* (TRITD3Bv1G280530.1) was also BLAST searched against the wheat reference genome sequences to identify common wheat homologue of *TdDof*.

Haplotype graphical genotypes and boxplot distributions were estimated involving 3B linkage maps, genotypic data and stem solidness score of recombinant lines, parental and check cultivars belonging to ‘Sadash/P2711’ and ‘AAC Innova/P2711’ populations using software package GGT 2.0 (Van Berloo, 2008), and R packages ggpubr (Kassambara, 2016) and rstatix (Kassambara, 2020).

Statistically significant differences among loci and their combinations were calculated by pairwise Games-Howell test using packages ggstatsplot (Patil, 2021) and tidyverse (Wickham et al., 2019) in R (version 4.0.3) (R Core Team, 2013).

## Results and discussion

### Phenotypic observations: Solid stem expression was high in doubled haploid lines

**Figure 1:**
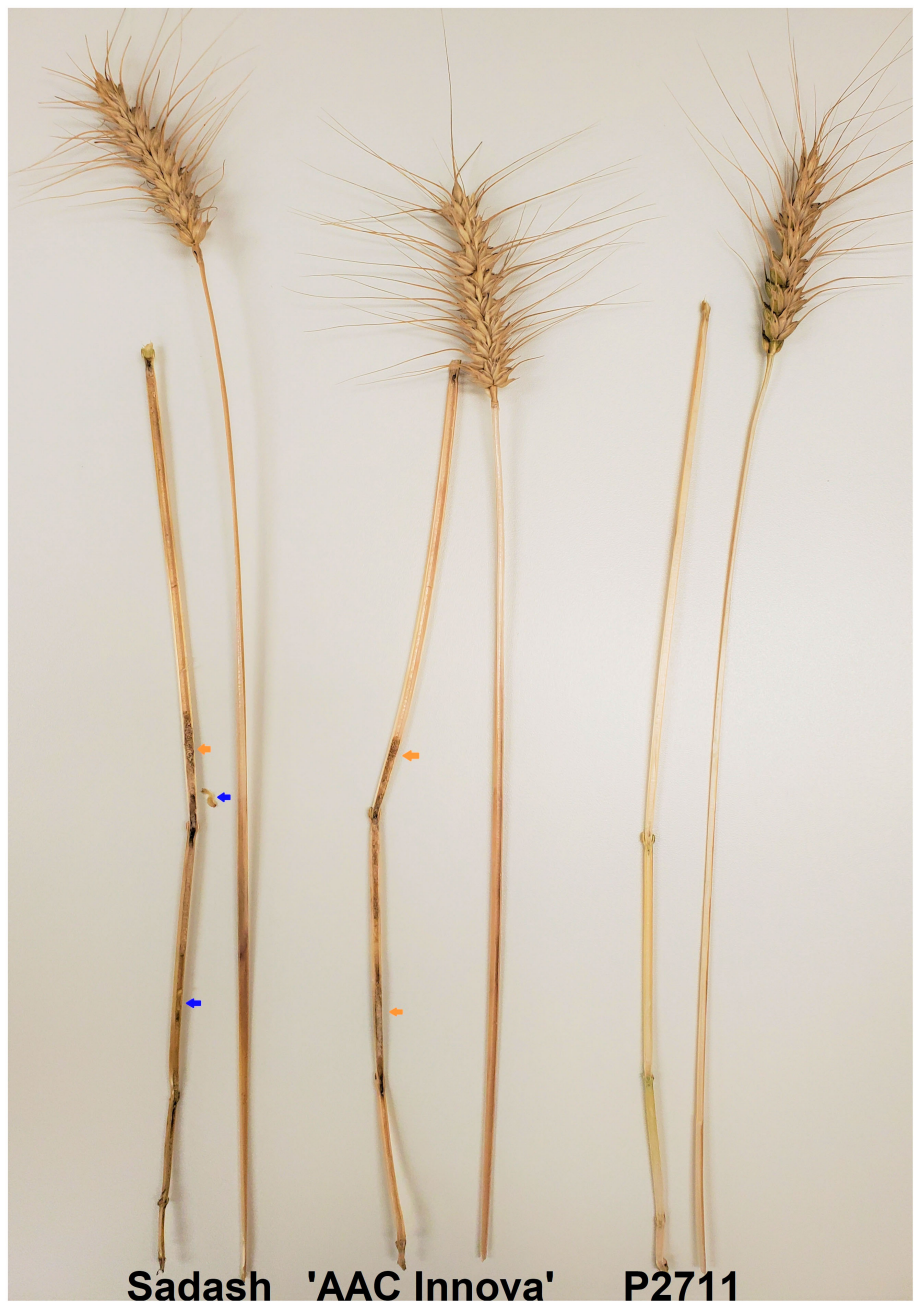
A representative picture of longitudinal dissection of stems (main tillers) of the three parental cultivars. Hollow stem cultivars Sadash and ‘AAC Innova’ are respectively shown on the left-hand side and in the middle while solid stem cultivar P2711 is on the right-hand side. Wheat stem sawfly (WSS) larvae detected in the stem of Sadash are shown by blue arrows while the girdled section filled with larva frass in both hollow stem cultivars Sadash and ‘AAC Innova’ are shown by orange arrows. All internodes of the stem of P2711 are completely filled with pith and have no sign of WSS damage. The picture was taken from mature plant stems collected from an irrigated field in Lethbridge, AB on September 1, 2022.

**Figure 2:**
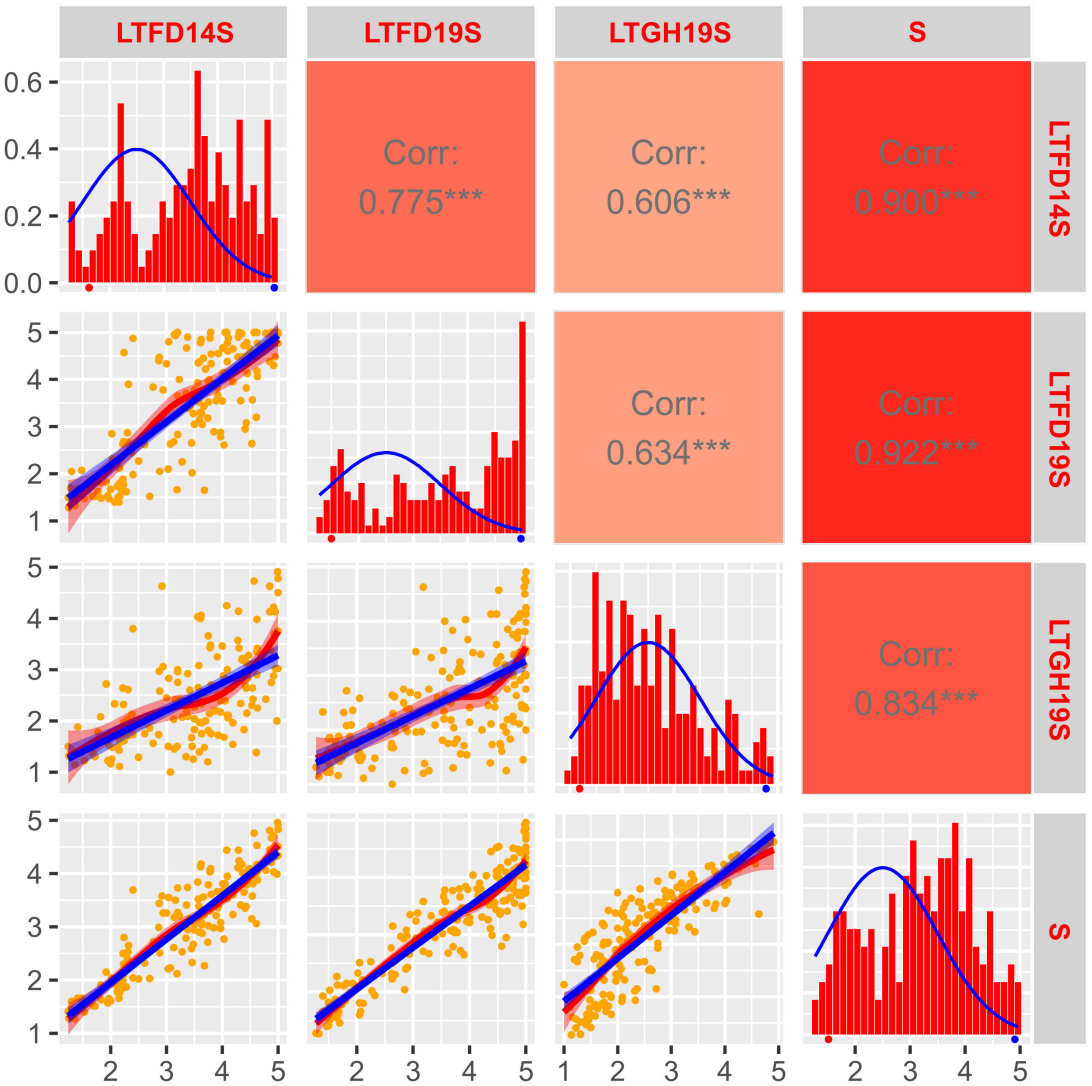
Frequency distribution and correlation scatterplots for solid stem rating of doubled haploid population ‘Sadash/P2711’. Frequency distribution histograms with distribution curve (blue line) for solid stem score of DHs grown at Lethbridge, Alberta in the field in 2014 (LTFD14S) and 2019 (LTFD19S) and greenhouse in 2019 (LTGH19S), as well as pooled data (average of all three environments; S) are shown on main diagonal. The means of the parental genotypes, Sadash (hollow-stemmed) and P2711 (solid-stemmed), are indicated by red and blue dots, respectively, beneath the frequency distribution plots. Scatterplots with regression lines, linear (blue) and exponential (red), for each environment are shown on the left side of the main diagonal. Orange dots on scatterplots represent the solid stem score of DHs. Correlation coefficients (*r*) between each pair of environments, and each environment and the pooled data, are displayed on the right side of the main diagonal. Color intensity (light red to dark red) on *r* boxes indicates the depth of association between environments under evaluation. ***p ≤ 0.001.

**Figure 3:**
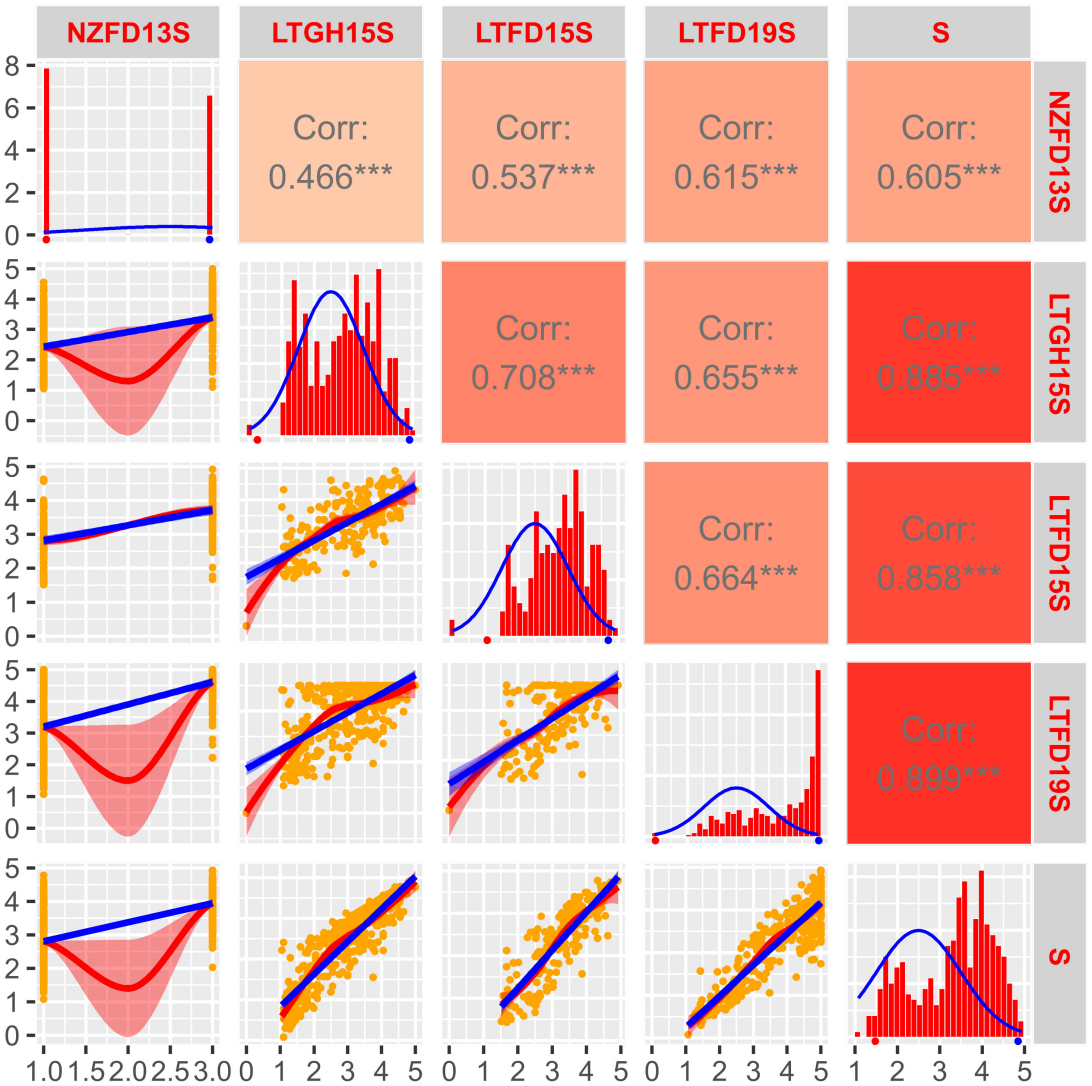
Frequency distribution and correlation scatterplots for solid stem rating of doubled haploid population ‘AAC Innova/P2711’. Frequency distribution histograms with distribution curve (blue line) for solid stem score of DHs grown in New Zealand field trial in 2013 (NZFD13S), at Lethbridge, Alberta in the greenhouse in 2015 (LTGH15S), the field in 2015 (LTFD15S) and 2019 (LTFD19S), as well as pooled data (average of all three environments; S) are shown on main diagonal. The means of the parental genotypes ‘AAC Innova’ (hollow-stemmed) and P2711 (solid-stemmed) are indicated by red and blue dots, respectively, beneath the frequency distribution plots. Scatterplots with regression lines, linear (blue) and exponential (red), for each environment are shown on the left side of the main diagonal. Orange dots on scatterplots represent the solid stem score of DHs. Correlation coefficients (*r*) between each pair of environments, and each environment and the pooled data, are displayed on the right side of the main diagonal. Color intensity (light red to dark red) on *r* boxes indicates the depth of association between environments under evaluation. ***p ≤ 0.001.

Phenotypic data analysis revealed substantial phenotypic variability for expression of stem solidness among the parents, check cultivars and DH lines across all environments for ‘Sadash/P2711’ and ‘AAC Innova/P2711’ populations (**Figures 1**–**3**; **Supplementary Table S1**). Expression of stem solidness was extremely low in the ‘AAC Cameron/P2711’ RIL population across environments, perhaps due to one or more reasons such as environmental effects (Weiss and Morrill, 1992; Cook et al., 2004), the residual heterozygosity present in RILs (Platt, 1941; Lanning et al., 2006), and unintended selection occurred during early generations. Thus, the phenotypic data of this population was not utilized for further analysis, however, genotypic data and the genetic map (Farzand et al., 2022) generated from ‘AAC Cameron/P2711’ at an advanced generation were utilized for the generation of chromosomes 3B and 3D specific consensus maps.

The ANOVA of solid stem score for both ‘Sadash/P2711’ and ‘AAC Innova/P2711’ populations revealed highly significant effects of genotypes as well as environments but not of genotype x environment interaction (**Supplementary Table S2**). The estimated broad-sense heritability (*H^2^*) values were also strong (0.78 for ‘Sadash/P2711’ and 0.69 for ‘AAC Innova/P2711’). The LS mean of solid stem rating values for P2711 was 4.92 or ~5.0 across environments, while it was ~1.5 for Sadash and ~1.6 for ‘AAC Innova’. The population mean values of solid stem rating ranged from 2.95 in LTGH19 to 3.17 in LTFD19 for the ‘Sadash/P2711’ population and 3.03 in LTGH15 to 3.22 in LTFD15 for the ‘AAC Innova/P2711’ population. Histograms showed either slightly skewed distribution towards more excellent stem solidness or binomial distribution for both populations across environments, except Lethbridge 2019 greenhouse experiments (LTGH19) for the ‘Sadash/P2711’ population and New Zealand, 2013 field trial (NZFD13) for ‘AAC Innova/P2711’ population (**Figures 2**, **3**). Additionally, a broader range of genotypes was observed across environments in both populations (**Figures 2**, **3**). Strong genotypic effect and high heritability of pith/stem solidness expression across environments along with skewed (towards more excellent solidness)/binomial distribution in both populations indicate the role of P2711 derived major solid stem genes/QTLs and no evidence for transgressive segregation (Lanning et al., 2006). However, a significant environmental effect also suggests the involvement of some minor and unstable QTLs.

Correlation coefficients (*r*) for the solid stem rating between any pair of environments were quite high and highly significant in the ‘Sadash/P2711’ population (**Figure 2**) and moderate to very high but highly significant in the ‘AAC Innova/P2711’ population (**Figure 3**). For the ‘Sadash/P2711’ population, Lethbridge 2019 greenhouse experiment showed relatively lower correlations with other environments (**Figure 2**). Similarly, the 2013 Lincoln, New Zealand experiment showed relatively lower correlations with other environments for the ‘AAC Innova/P2711’ population (**Figure 3**). Lower correlations between any environment pair than the broad-sense heritability values (0.78 for ‘Sadash/P2711’ and 0.69 for ‘AAC Innova/P2711’ populations) further suggest the existence of strong environmental influence (Gondo et al., 2007) on stem solidness which has also been reported previously for solid-stem phenotype in common wheat DH population ‘Rampart/Jerry’ (Cook et al., 2004). Irrespective of environmental effects, DH solid stem data was of high quality and found suitable for identifying QTLs in these populations.

### High-density linkage maps

**Figure 4:**
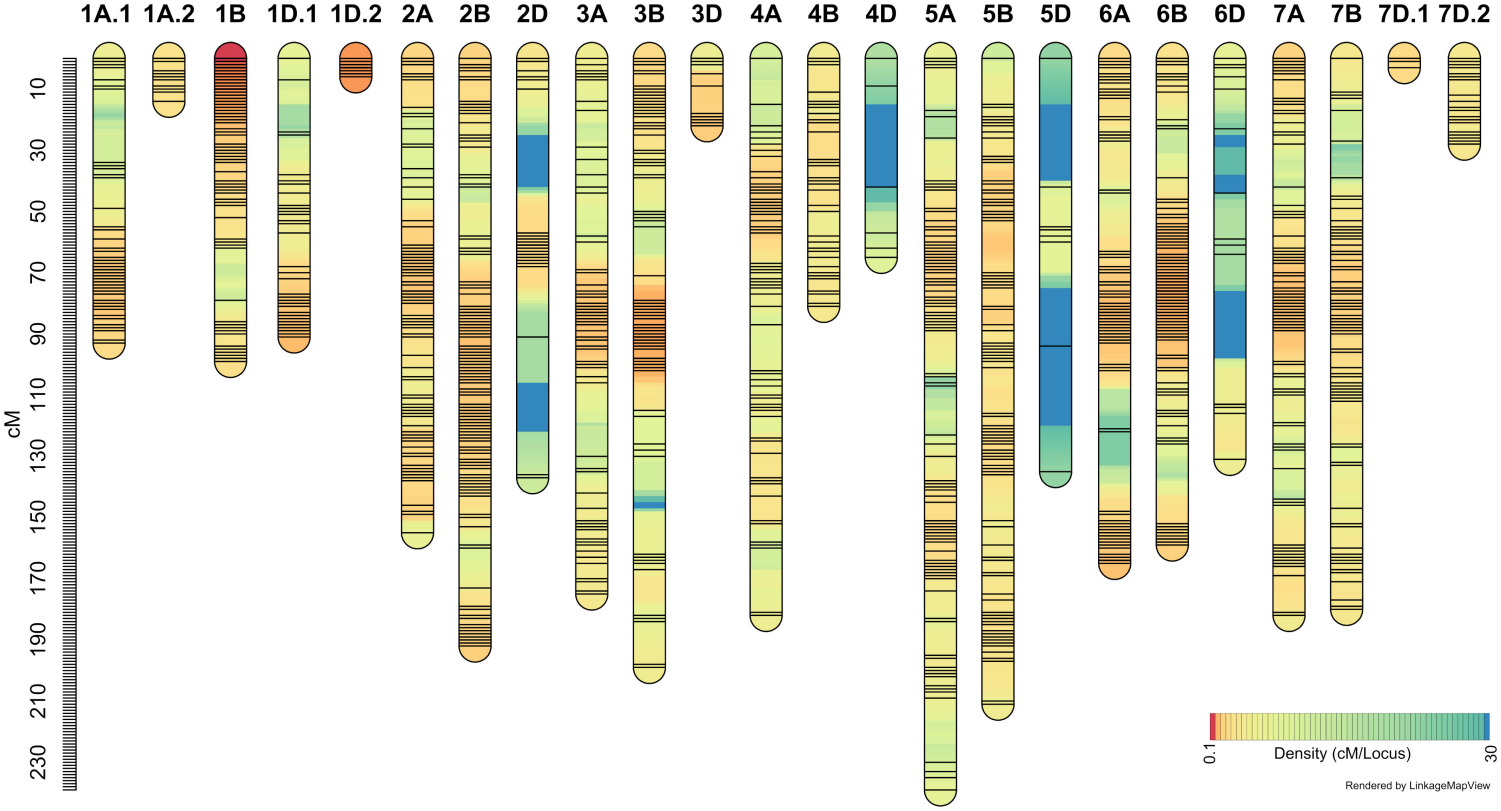
‘Sadash/P2711’ linkage map constructed using single nucleotide polymorphism (SNP) markers from 15K wheat SNP array.

**Figure 5:**
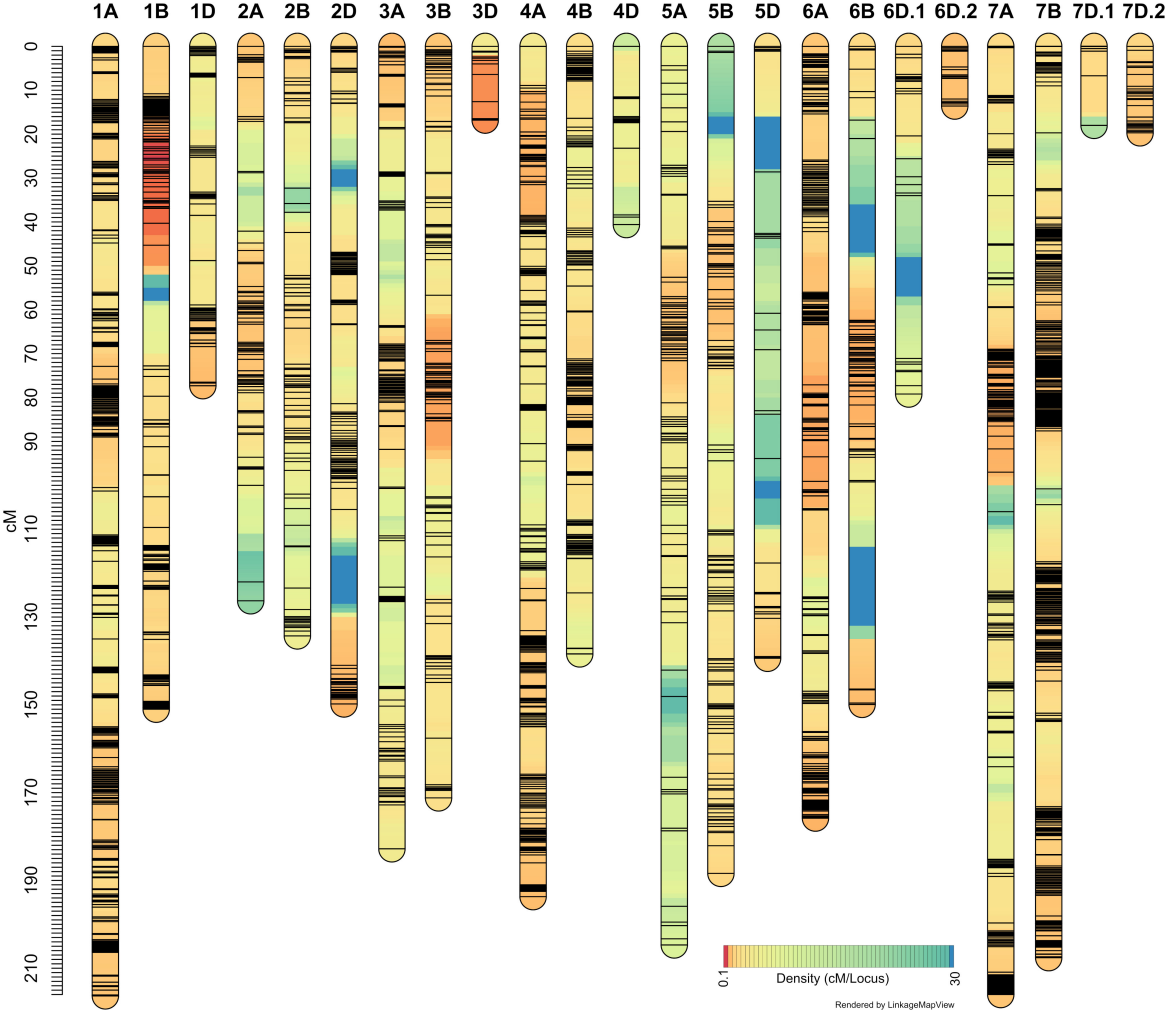
‘AAC Innova/P2711’ linkage map constructed using single nucleotide polymorphism (SNP) markers from 90K wheat SNP array.

It may be recalled that the ‘Sadash/P2711’ population was genotyped using Illumina 15K Wheat SNP array while ‘AAC Innova/P2711’ using Illumina 90K Wheat SNP array. A total of 4,959 SNP markers were found polymorphic between two parents of the ‘Sadash/P2711’ population, of which, 4,837 were mapped to 24 linkage groups in that population (**Figure 4**; **Supplementary Table S3**). A total of 13,425 SNPs were found polymorphic between two parents of the ‘AAC Innova/P2711’ population, of which, 8,930 high-quality SNPs were successfully mapped to 23 linkage groups (**Figure 5**; **Supplementary Table S4**). Map of ‘AAC Cameron/P2711’ has been described and published previously (Farzand et al., 2022). Briefly, a total of 8,915 markers were incorporated into 29 linkage groups constructed using this population.

‘Sadash/P2711’ map spanned a map distance of 2953.9 cM (on average 123.08 cM per LG or 140.66 cM per chromosome) with an average marker interval of 2.05 cM and an average SNP distance of 0.61 cM (**Figure 4**; **Supplementary Table S5**). Similarly, the ‘AAC Innova/P2711’ map spanned a map distance of 3010.65 cM (on average 130.9 cM per LG or 143.36 cM per chromosome) with an average marker interval of 1.12 cM and an average SNP distance of 0.34 cM (**Figure 5**; **Supplementary Table S5**). The total map lengths of linkage groups for sub-genomes A, B, and D were 1208.5, 1124.2, and 621.2, respectively, for the ’Sadash/P2711’ population; similarly, sub-genomes A, B, and D map lengths were 1315.65, 1140.68, and 554.32, respectively, for the ‘AAC Innova/P2711’ population. These results showed that where genome A had the highest SNP coverage in both populations, which is similar to genome B, genome D had the lowest coverage with almost half of either A or B genome. In both linkage maps, the D-genome chromosomes are significantly underrepresented in comparison with the A- and B-genome chromosomes. We also observed uneven distribution of SNPs along the linkage groups and the formation of SNP clusters in certain regions which led to the formation of large gaps in many linkage groups, particularly in some sub-genome D-specific chromosomes in both populations (**Figures 4**, **5**). These variations are perhaps due to (i) the lower number of analyzed markers on genome D or other genomes, (ii) similarity of genomic regions where the parents are identical by descent, and (iii) exome specificity of the mapped SNPs (Dhariwal et al., 2018). Similar results have also been reported previously (Dhariwal et al., 2018; Dhariwal et al., 2020).

On the other hand, the highest number of SNPs were mapped to wheat homoeologous group 1 chromosomes in both populations (863 and 2102 SNPs in ‘Sadash/P2711’ and ‘AAC Innova/P2711’, respectively) but the lowest number to group 4 chromosomes (335 SNPs) in ’Sadash/P2711’ population and group 5 chromosomes (752) in ‘AAC Innova/P2711’ population. Likewise, the highest marker densities were reported on group 1 chromosomes in both populations (0.35 and 0.21 cM/SNP, respectively in the ‘Sadash/P2711’ and ‘AAC Innova/P2711’ populations). Interestingly, a total of 74 SNPs belonging to the ‘Sadash/P2711’ population and 317 to the ‘AAC Innova/P2711’ population mapped in this study represent new mapped markers (**Supplementary Tables S3, S4**) which were not present on either wheat consensus map (Wen et al., 2017) or previous wheat maps used for solid stem studies (Nilsen et al., 2017).

Where the distributions of mapped markers over the linkage groups of all populations are satisfactory, the map length and the number of markers of linkage groups constructed in this study are in agreement with the previous genetic maps (Wang et al., 2014; Wen et al., 2017; Dhariwal et al., 2018; Dhariwal et al., 2020; Farzand et al., 2021; Farzand et al., 2022). Moreover, maps constructed in this study cover all 21 wheat chromosomes and provides enough coverage to dissect the genetic variation of stem solidness present in these populations.

### Two major solid stem loci derived from P2711

**Figure 6:**
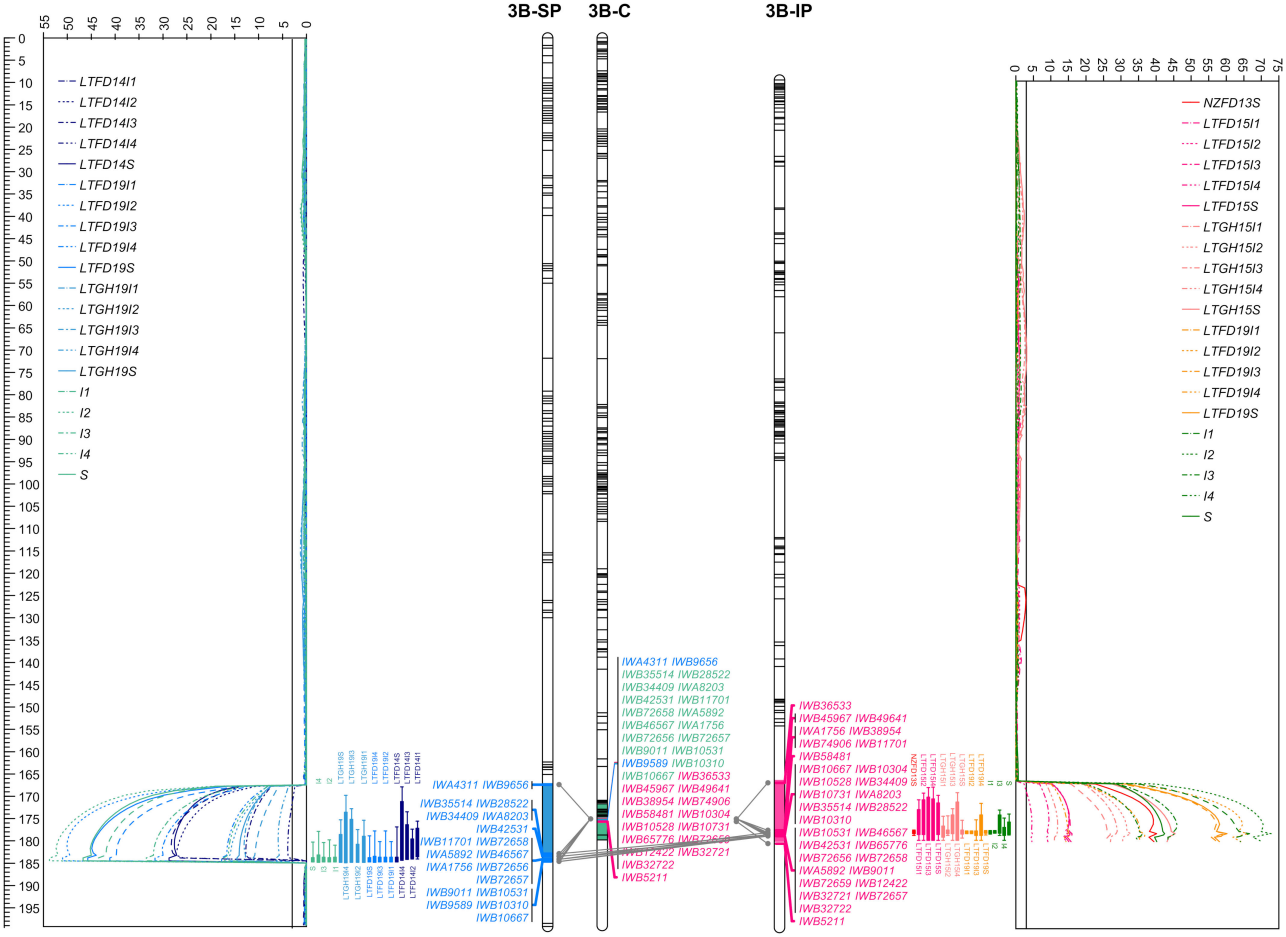
The linkage maps of chromosome 3B deciphering the logarithm of odds (LOD) curve plots for major solidness (stem, S and internode, I) quantitative trait locus (QTL) *QSst.lrdc-3B* detected using composite interval mapping from ‘Sadash/P2711’ (3B-SP) and ‘AAC Innova/P2711’ (3B-IP) doubled haploid populations in each of the trial environments and pooled data along with a chromosome 3B consensus map (3B-C) in middle. Positions of markers are shown as lines on linkage group bars and a scale in cM on the left of the graph. SNP markers common between the two linkage groups and the consensus map are shown by SNP IDs in green font, while markers common between a population map and the consensus map are shown in blue (for ‘Sadash/P2711’ markers) or dark pink (for ‘AAC Innova/P2711’ markers) color. Relationships among linkage groups and consensus map are shown using gray color connections for each common marker. QTL intervals are shown using a different colored segment of linkage group bars, while QTL confidence intervals for each environment/trait combination are shown using different colored bars against LOD curve plots. LTFD14(I1-I4, and S), LTFD19(I1-I4, and S), LTGH19(I1-I4, and S), and single letter (and numerals) I1 to I4 and S represent QTL detected in field experiments in 2014, 2019, greenhouse experiment in 2019 and pooled data obtained from Lethbridge, Alberta location for whole stem and culm internodes I1 (uppermost) to I4 (lowermost) ratings of doubled haploid lines of ‘Sadash/P2711’ population. Similarly, NZFD13S, LTFD15(I1-I4, and S), LTGH15(I1-I4, and S), LTFD19(I1-I4, and S), and single letters (and numerals) I1 to I4 and S represent QTL detected in field experiments in New Zealand in 2013, Lethbridge field trials in 2015, Lethbridge greenhouse experiments in 2015, Lethbridge field trial in 2019 and pooled data obtained from Lethbridge, Alberta location for the whole stem and culm internodes I1 (uppermost) to I4 (lowermost) ratings of doubled haploid lines of ‘AAC Innova/P2711’ population. Straight lines across the LOD plot represent the LOD significance thresholds.

**Figure 7:**
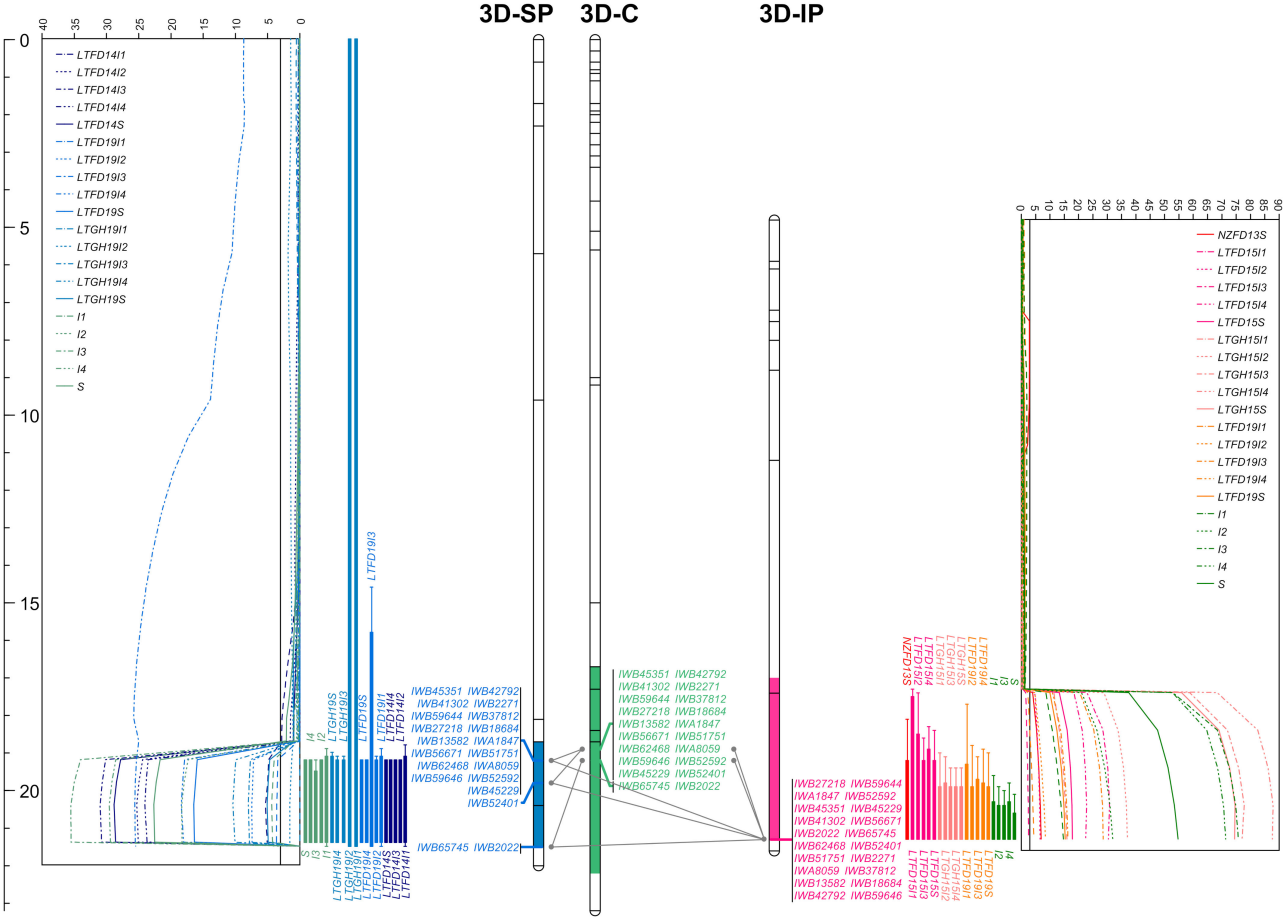
The linkage maps of chromosome 3D deciphering the logarithm of odds (LOD) curve plots for major solidness (stem, S and internode, I) quantitative trait locus (QTL) *QSst.lrdc-3D* detected using composite interval mapping from ‘Sadash/P2711’ (3D-SP) and ‘AAC Innova/P2711’ (3D-IP) doubled haploid populations in each of the trial environments and pooled data along with a chromosome 3D consensus map (3D-C) in middle. Positions of markers are shown as lines on linkage group bars and a scale in cM on the left of the graph. SNP markers common between the two linkage groups and the consensus map are shown by SNP IDs in green font, while markers common between a population map and the consensus map are shown in blue (for ‘Sadash/P2711’ markers) or dark pink (for ‘AAC Innova/P2711’ markers) color. Relationships among linkage groups and consensus map are shown using gray color connections for each common marker. QTL intervals are shown using a different colored segment of linkage group bars, while QTL confidence intervals for each environment/trait combination are shown using different colored bars against LOD curve plots. LTFD14(I1-I4, and S), LTFD19(I1-I4, and S), LTGH19(I1-I4, and S), and single letter (and numerals) I1 to I4 and S represent QTL detected in field experiments in 2014, 2019, greenhouse experiment in 2019 and pooled data obtained from Lethbridge, Alberta location for whole stem and culm internodes I1 (uppermost) to I4 (lowermost) ratings of doubled haploid lines of ‘Sadash/P2711’ population. Similarly, NZFD13S, LTFD15(I1-I4, and S), LTGH15(I1-I4, and S), LTFD19(I1-I4, and S), and single letters (and numerals) I1 to I4 and S represent QTL detected in field experiments in New Zealand in 2013, Lethbridge field trials in 2015, Lethbridge greenhouse experiments in 2015, Lethbridge field trial in 2019 and pooled data obtained from Lethbridge, Alberta location for the whole stem and culm internodes I1 (uppermost) to I4 (lowermost) ratings of doubled haploid lines of ‘AAC Innova/P2711’ population. Straight lines across the LOD plot represent the LOD significance thresholds.

Whole genome QTL analyses identified that solid stem expression in both populations was mostly modulated by two major and stable loci mapped to chromosomes 3B (*QSst.lrdc-3B*) and 3D (*QSst.lrdc-3D*) in addition to some minor loci (**Table 1**; **Figures 6**, **7**; **Supplementary Table S6**) as well as epistasis effect (**Supplementary Table S7**). The two major QTLs are discussed first in greater detail followed by others below.

**Table 1:** Summary of quantitative trait loci (QTLs) identified for stem solidness in ‘Sadash/P2711’ and ‘AAC Innova/P2711’ populations.

QTL Name	Chr	Pos.	Interval_max_	LOD_max_	Additive effect_max_	%*R^2^*_max_		Environment(s) CIM	S and/or Int.	Closest marker and position			Donor allele
						S	Int.			SNP ID	cM	bp	
**‘Sadash/P2711’ – major and stable QTLs**													
*QSst.lrdc-3B^ 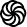 ^*	3B	183.9	167.1 – 184.7	53.8	-1.33	64.0	77.0	LTFD14, LTFD19, LTGH19, C	S, I1 – I4	IWB11701	184.1	843247610	P2711
*QSst.lrdc-3D^ 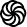 ^*	3D	20.4	18.7 – 21.5	35.6	-1.56	40.0	52.0	LTFD14, LTFD19, LTGH19, C	S, I1 – I4	TG3069*	20.4	603821560	P2711
**‘Sadash/P2711’ – minor QTLs**													
*QSst.lrdc-1D*	1D.1	5.3	1.2 – 10.1	3.4	-0.24	5.0	5.0	LTGH19	S, I3	IWA3753	7.8	3051756	P2711
*QSst.lrdc-2A^ 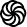 ^*	2A	53.9	42.2 – 69.7	10.4	-0.43	6.0	11.0	LTFD14, LTFD19, C	S, I1 – I3	IWB11289	53.9	64102080	P2711
*QSst.lrdc-4A.1*	4A	3.4	0.0 – 15.1	6.2	-0.32	4.0	6.0	LTFD14	S, I2	IWB8008	0.0	3365864	P2711
*QSst.lrdc-4A.2^ 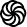 ^*	4A	50.0	43.1 – 52.8	3.7	-0.15	3.0	2.0	C	S, I2	IWB46934	50.0	487314533	P2711
*QSst.lrdc-5A.1^ 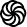 ^*	5A	75.7	73.0 – 91.8	4.0	-0.25	2.0	6.0	LTGH19, C	S, I2	IWB36264	76.4	485652482	P2711
*QSst.lrdc-5A.2*	5A	166.9	140.5 – 172.7	6.1	0.32	9.0	9.0	LTGH19, C	S, I2 – I4	IWB47006	166.8	609465091	HP
*QSst.lrdc-6B*	6B	93.8	65.0 – 108.2	4.6	0.28	3.0	8.0	LTFD14, LTGH19	S, I1, I3	IWB60190	92.7	657946476	HP
*QSst.lrdc-7B*	7B	8.1	4.9 – 14.3	3.2	0.15	2.0	na	C	S	IWB6919	11.9	3541540	HP
**‘AAC Innova/P2711’ – major and stable QTLs**													
*QSst.lrdc-3B^ 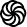 ^*	3B	168.8	157.0 – 170.6	75.0	-1.18	52.0	58.0	NZFD13, LTGH15, LTFD15, LTFD19, C	S, I1 – I4	IWA1756	168.7	843250756	P2711
*QSst.lrdc-3D^ 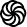 ^*	3D	16.5	12.2 – 16.5	88.0	-1.00	46.0	56.0	NZFD13, LTGH15, LTFD15, LTFD19, C	S, I1 – I4	IWB52401	16.5	611013692	P2711
**‘AAC Innova/P2711’ – minor QTLs**													
*QSst.lrdc-1B*	1B	33.2	32.9 – 33.9	3.5	-0.12	1.0	na	LTGH15	S	IWB26730	33.2	10355943	P2711
*QSst.lrdc-2A.1*	2A	16.1	14.8 – 16.2	3.2	-0.16	2.0	na	LTFD19	S	IWB10662	16.0	36651065	P2711
*QSst.lrdc-2A.2^ 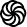 ^*	2A	58.7	46.3 – 60.2	7.5	-0.21	3.0	3.0	LTGH15, C	S, I1 – I4	IWA3194	58.6	76368584	P2711
*QSst.lrdc-2B*	2B	56.3	47.9 – 81.4	3.7	0.97	na	4.0	LTGH15, LTFD15, LTFD19	I2 – I3	IWA5410	56.1	52005347	HP
*QSst.lrdc-2D*	2D	141.1	135.9 – 142.7	3.6	-0.52	na	5.0	LTFD15	I1	IWB64223	141.0	640832761	P2711
*QSst.lrdc-4A^ 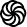 ^*	4A	24.3	21.6 – 34.8	4.2	-0.17	na	3.0	LTGH15	I1	IWB1356	24.6	345813273	P2711
*QSst.lrdc-4D*	4D	16.9	13.7 – 28.3	3.8	-0.24	4.0	6.0	LTFD15	S, I1	IWB12286	16.8	455964578	P2711
*QSst.lrdc-5A^ 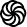 ^*	5A	67.0	53.1 – 72.8	4.0	-0.16	1.0	2.0	LTGH15	S, I2 – I3	IWB71951	67.0	482845859	P2711
*QSst.lrdc-5B*	5B	55.5	52.3 – 57.5	6.4	-0.24	2.0	2.0	LTGH15	S, I2 – I4	IWB72268	55.4	459560328	P2711
*QSst.lrdc-5D*	5D	60.9	37.1 – 74.3	5.3	0.20	2.0	4.0	LTGH15	S, I1, I3	IWB63558	60.8	465885796	HP
*QSst.lrdc-7B*	7B	14.0	8.6 – 19.2	4.3	-0.17	na	2.0	LTGH15	I3	IWB51995	9.0	13398372	P2711

Chr, chromosome; Pos., linkage map position (cM) belonging to highest LOD peak of QTL in any single environment (NZFD13, New Zealand Field Trial 2013; LTFD14, Lethbridge Field Trial 2014; LTGH15, Lethbridge Greenhouse Experiment 2015; LTFD15, Lethbridge Field Trial 2015; LTGH19, Lethbridge Greenhouse Experiment 2019; and LTFD19, Lethbridge Field Trial 2019) or pooled/combined (C) data of stem/internode solidness; Interval_max_, QTL interval (cM) calculated using markers identified based on composite interval mapping (CIM) in all the environments for stem and/or internode solidness dataset(s); LOD, logarithm of the odds score for a detected QTL; LOD_max_, Additive effect_max_ and %R^2^_max_, highest score reported in any single environment or pooled/combined data; %*R^2^*, percent phenotypic variation explained by a detected QTL; S, stem solidness; Int., internode solidness; S and/or Int., QTL detected for stem solidness (S) and/or internode (I) score; I1, internode 1/peduncle; I2, internode 2; I3, internode 3; and I4, internode 4/lowest internode scored; SNP ID, single nucleotide polymorphism marker identity based on 90K Wheat SNP array or 15K Wheat SNP array (*); cM, centimorgan; bp, base pairs; cM and bp positions are based on ‘Sadash/P2711’ or ‘AAC Innova/P2711’ linkage map and IWGSC RefSeq v.2.1 physical map/genome, respectively; ^
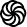
^QTLs common in both populations; Donor allele: P2711 – solid stem parent, HP – hollow stem parent Sadash in ‘Sadash/P2711’ population and ‘AAC Innova’ in ‘AAC Innova/P2711’ population.

*QSst.lrdc-3B* mapped on the distal region of the long arm of chromosome 3B was detected in both populations across all environments as well as in pooled data. Moreover, while it was detected using whole stem solidness data, it was also detected for all 4 upper internodes rated in this study. Using population-specific maps, we identified that this QTL was derived from the solid-stem parent P2711 and explained up to 64.0% and 52% of the phenotypic variation (PV) for whole stem solidness and 77.0% and 58.0% of PV for internodes in ‘Sadash/P2711’ and ‘AAC Innova/P2711’ populations, respectively (**Table 1**). The P2711 allele at this locus had an internode additive effect up to 1.33 in ‘Sadash/P2711’ population and up to 1.18 in the ‘AAC Innova/P2711’ population (**Table 1**) which increased stem solidness by around 24.0 - 27.0%. Major QTLs have been previously repeatedly mapped to the same overlapping region as *QSst.lrdc-3B* in common wheat cultivars (e.g., Canadian and US cvs. Choteau, Lillian Rescue, Rampart and Scholar all derived from Portuguese landrace S-615 and cv. with unknown solid stem resource like US cv. Conan) and to the distal region of chromosome 3B of durum wheat (e.g., Canadian cvs. derived from German cv. Biodur and South African landrace Golden Ball) (Cook et al., 2004; Lanning et al., 2006; Houshmand et al., 2007; Sherman et al., 2010; Talbert et al., 2014; Varella et al., 2015; Nilsen et al., 2017). Similar to P2711-derived 3B QTL, previously identified QTLs in this region were also reported to explain as much as 76% (Cook et al., 2004) to 78% (Nilsen et al., 2017) of phenotypic variation for whole stem rating in common wheat. Cook et al. (2017) demonstrated that all WSS-resistant common wheat North American cultivars, except ‘Conan’, evaluated in their study had the same haplotype at the major 3BL locus derived from ‘S-615’ but this haplotype was not found in the solid-stem tetraploid landraces. Recently, using more detailed analysis, Nilsen et al. (2020) reported that a single gene is responsible for stem solidness at the 3B locus across durum and common wheat cultivars, and perhaps it originated from a common genetic progenitor, either pre- or post-domestication. Although the exact source of stem solidness at the 3B locus in P2711 is unknown, P2711 also most likely derived its stem solidness from the same progenitor as other durum and common wheat cvs. since both P2711 and the durum landrace ‘Golden Ball’ originated from South Africa.

*QSst.lrdc-3D* is another very strong major and stable QTL derived from the male parent P2711 and explained up to 40.0% and 46.0% of the phenotypic variation for whole stem solidness and up to 52.0% and 56.0% of PV for internodes in ‘Sadash/P2711’ and ‘AAC Innova/P2711’ populations, respectively (**Table 1**). Like *QSst.lrdc-3B*, *QSst.lrdc-3D* was also mapped on the distal region of the long arm of its chromosome 3D and detected in both populations across all environments as well as in pooled data for whole stem solidness and 4 internodes rating (**Table 1**; **Figure 7**; **Supplementary Table S6**). The P2711 allele at this locus had an internode additive effect up to 1.56 in ‘Sadash/P2711’ and up to 1.00 in the ‘AAC Innova/P2711’ population (**Table 1**) which increased stem solidness by around 20.0 - 31.0%. A secondary QTL (*Qss.msub-3DL*) has also been previously mapped to chromosome arm 3DL in common wheat cultivar Choteau (Lanning et al., 2006). Interestingly, both Choteau and P2711 are hard red spring wheat lines and possess the solid stem locus on the distal region of chromosome arm 3DL. However, while Choteau QTL explained only 31.0% of phenotypic variation, Choteau also does not share its immediate parentage with P2711. Moreover, we found that *Qss.msub-3DL*’s closely associated microsatellite marker *Xgwm645* (Lanning et al., 2006) is physically located proximal (chromosome position 454946571) to *QSst.lrdc-3D*’s closely linked SNP marker IWB52401 (chromosome position 609087492) on chromosome 3D of cv ‘CDC Landmark’. Since the original source of stem solidness in P2711 is not known and differences in positions of *QSst.lrdc-3D* and *Qss.msub-3DL* may be due to shifts in QTL positions as a result of differences in phenotyping or genotyping, it remains to be determined whether *QSst.lrdc-3D* and *Qss.msub-3DL* are the same or represent different loci. However, the differences in explained PV of *QSst.lrdc-3D* and *Qss.msub-3DL* indicate that the P2711 most likely carries at least a different allele at this locus. Future haplotype study of two genotypes, Choteau and P2711, using all closely linked and gene-based markers of *QSst.lrdc-3D* and *Qss.msub-3DL* may help in establishing a definitive relationship between the two identified QTLs.

To identify additional markers at these QTLs as well as markers common between two populations, we constructed consensus maps for chromosomes 3B and 3D using the mapping data of all three individual populations used in this study. Chromosome 3B consensus map spans a total map length of 179.836 cM, while the 3D map spans 23.147 cM (**Supplementary Tables S8, S9**). These maps have higher resolution (map density: 0.16 and 0.04 cM/SNP, respectively, for chromosomes 3B and 3D) and higher marker coverage than individual population-specific maps. QTL analysis using these maps identified the same solid stem loci as detected using population-specific maps but with higher confidence and additional markers (**Supplementary Table S10**). We further observed that *QSst.lrdc-3B* explained up to 93.0% of phenotypic variation for whole stem solidness and 78.0% for internodes, while *QSst.lrdc-3D* explained up to 46.0% of phenotypic variation for whole stem solidness and 67.0% for internodes (**Supplementary Table S10**). We also found several common markers between ‘Sadash/P2711’ and ‘AAC Innova/P2711’ populations for each of the two chromosomes, 3B and 3D (**Figures 6**, **7**). Selection for the solid stem alleles on chromosome arms 3BL and 3DL using these common markers will be more useful than population specific markers for the deployment of stem solidness in different genetic backgrounds to develop new cultivars.

#### *QSst.lrdc-3D* is homeologous to *QSst.lrdc-3B*

Synteny analysis of chromosomes 3B and 3D linkage maps of both populations with the 3B physical map of ‘CDC Landmark’, a Canadian solid stem wheat cultivar, showed the consistency of marker order between the genetic and physical maps except for a few SNPs (**Supplementary Figure S1**). A similar syntenic relationship was observed when we compared the 3B and 3D linkage maps of both populations with the 3D physical map of ‘CDC Landmark’ (**Supplementary Figure S2**). Particularly, the order of SNP markers on 3B linkage groups of both populations and 3B and 3D physical maps of ‘CDC Landmark’ was highly consistent (**Supplementary Figure S2**). However, we observed that a lower recombination rate or similarity of 3D chromosomes of population parents in both crosses (‘Sadash/P2711’ and ‘AAC Innova/P2711’), which largely seems to be identical by descent, leads to the low-resolution mapping of markers on this chromosome. This also resulted in some discrepancies between the marker order of 3D linkage maps of two crosses and the physical map (**Supplementary Figure S2**). Interestingly, *QSst.lrdc-3B* and *QSst.lrdc-3D* from both populations overlap the same genomic region, which also possesses the wheat homologue of durum solid stem gene *TdDof*, on the 3B physical map of ‘CDC Landmark’ (**Supplementary Figure S1**). Similarly, both major solid stem loci identified in this study overlap the same genomic region on the 3D physical map of ‘CDC Landmark’ (**Supplementary Figure S2**) irrespective of minor marker order discrepancies. Our results indicate that both solid stem loci identified in this study are homeologous to each other.

#### Haplotype analysis located *QSst.lrdc-3B* to a 1.1 Mb region on chromosome 3B

**Figure 8:**
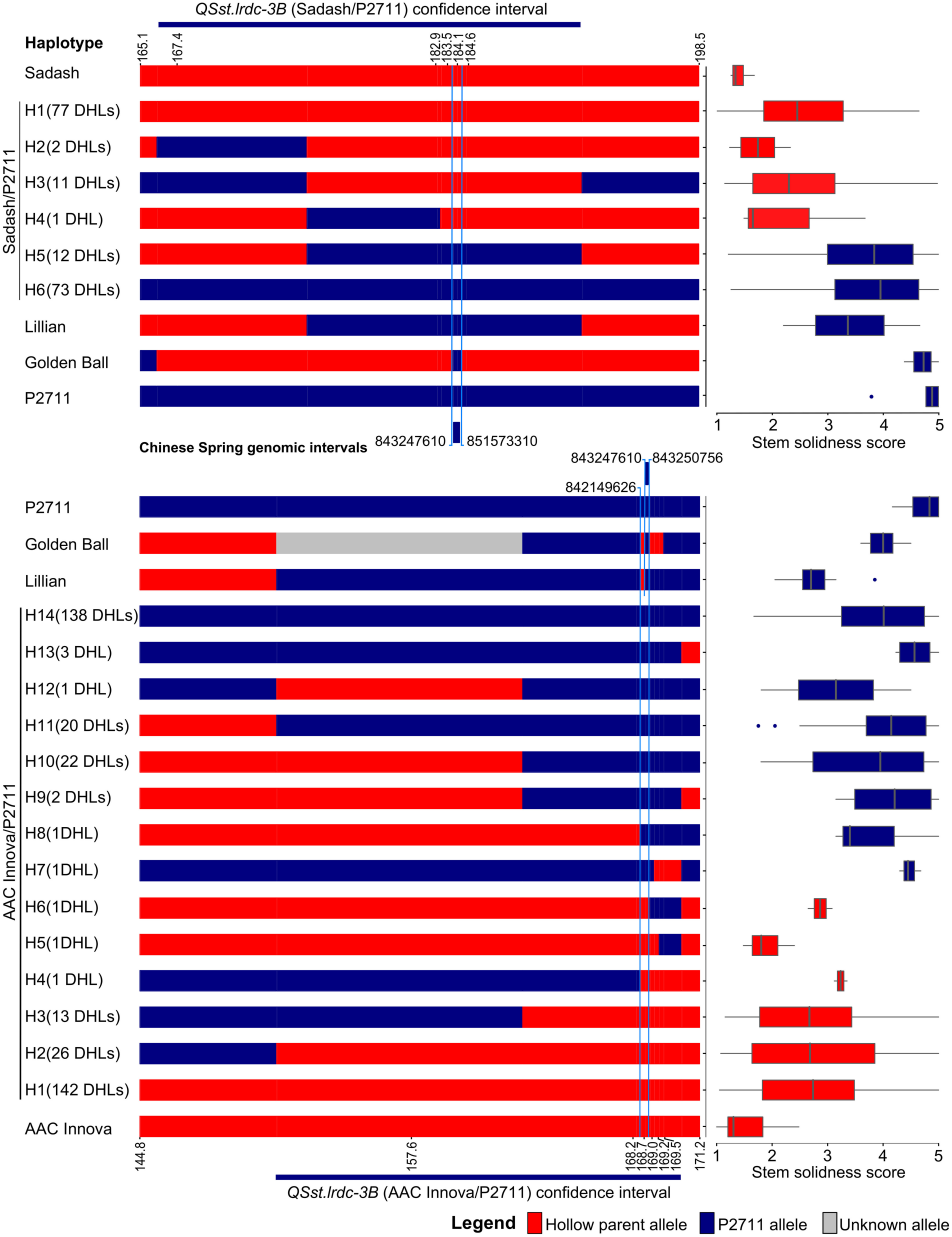
Graphical genotypes of wheat haplotypes (H) of doubled haploid lines (DHLs), hollow (Sadash and ‘AAC Innova’)- and solid (P2711)-stem parents, and solid-stem check cultivars (Lillian and ‘Golden Ball’) displaying recombination breakpoints in the QTL region *QSst.lrdc-3B* on linkage group 3B. Boxplots showing mean stem solidness score distribution across environments for respective haplotypes. QTL segments marked by blue color lines completely co-segregate with stem solidness.

Using haplotype analysis of the ‘Sadash/P2711’ population, we located *QSst.lrdc-3B* in a chromosomal region between 843.24 and 851.57 Mb on chromosome 3B of Chinese Spring (**Figure 8**). We further narrowed this region using haplotype data of the ‘AAC Innova/P2711’ population and cvs Lillian and ‘Golden Ball’, which located 3B solid stem underlying gene between 842.14 and 843.25 Mb on chromosome 3B (hereinafter referred to as ‘3B solid stem segment’) of Chinese Spring (**Figure 8**). We found that both Lillian and ‘Golden Ball’ share common alleles in this genomic region. Interestingly, *TaDof*, a homolog of the durum *TdDof* gene, was located outside (at 843.64 Mb) this region on chromosome 3B. SNP marker RAC875_c48860_106 (IWB58481), which was genetically mapped distal to this 1.1 Mb ‘3B solid stem segment’ on ‘AAC Innova/P2711’ 3B map, had an ‘AAC Innova’ type allele in cv. ‘Golden Ball’ and was located physically at 843.94 Mb on Chinese Spring chromosome 3B. Since, the position of recombination breakpoint between markers co-segregating in the ‘3B solid stem segment’ and the SNP marker RAC875_c48860_106 is not known due to genetic type of data, which may be anywhere between 843.25 and 843.94 Mb, it remains to be determined whether *TaDof* co-segregate with ‘3B solid stem segment’ or not.

### Minor solid-stem loci

In addition to above two major stable QTLs, we detected a number of minor QTLs on chromosomes 1B (‘AAC Innova/P2711’ population), 1D (‘Sadash/P2711 population’), 2A (both populations), 2B and 2D (‘AAC Innova/P2711’ population), 4A (both population), 4D (‘AAC Innova/P2711’ population), 5A (both populations), 5B and 5D (‘AAC Innova/P2711’ population), 6B (‘Sadash/P2711’ population) and 7B (both population) (**Table 1**).

Most of these minor loci explained <5% of phenotypic variation for whole stem solidness except *QSst.lrdc-1D*, *QSst.lrdc-2A* and *QSst.lrdc-5A.2* from the ‘Sadash/P2711’ population, which respectively explained 5%, 6% and 9% phenotypic variation. Moreover, most of these loci were detected in one environment/one environment with pooled data or just pooled data, except *QSst.lrdc-2A* and *QSst.lrdc-6B* from the ‘Sadash/P2711’ population and *QSst.lrdc-2B* from ‘AAC Innova/P2711’ population. Except for QTLs *QSst.lrdc-5A.2*, *QSst.lrdc-6B*, and *QSst.lrdc-7B* of ‘Sadash/P2711’ population and *QSst.lrdc-2B* and *QSst.lrdc-5D* of ‘AAC Innova/P2711’ population, solid stem allele at all loci were derived from P2711 in both populations. Solid stem alleles at remaining five loci were derived from the hollow-stemmed parent of respective population. On the other hand, *QSst.lrdc-2A* of ‘Sadash/P2711’ and *QSst.lrdc-2A.2* of ‘AAC Innova/P2711’ are supposed to be the same locus as they shared a common genomic location on the wheat reference genome (**Supplementary Tables S11, S12**). Similarly, *QSst.lrdc-4A.2* of ‘Sadash/P2711’ and *QSst.lrdc-4A* of ‘AAC Innova/P2711’ belongs to the same locus on chromosome 4A, in addition to *QSst.lrdc-5A.2* of ‘Sadash/P2711’ and *QSst.lrdc-5A* of ‘AAC Innova/P2711’ on chromosome 5A (**Supplementary Tables S11**, **S12**). These results indicate that the identified common QTLs can be utilized in different genetic backgrounds (Dhariwal et al., 2021) for improving solid stem as an adaptive trait. Minor QTLs, other than these common QTLs, were population specific. These unique loci can also be of regional utility (Dhariwal et al., 2021) for solid stem development in wheat.

Minor solid stem loci have been previously detected on chromosomes 1B, 2A, 3AL, 4A, 5A, 5B, 5D and 6B (Larson and Macdonald, 1959a; Sherman et al., 2010; Varella et al., 2015; Nilsen et al., 2017; Varella et al., 2019). Among these, 1B, 5A, 5B and 5D minor loci were detected in common or durum wheat (Larson and Macdonald, 1959a; Sherman et al., 2010; Varella et al., 2015), while 2A, 3AL, 4A and 6B loci were exclusively detected in durum wheat (Nilsen et al., 2017; Varella et al., 2019). While all these loci affect solid stem expression, two (1B and 3AL) of these loci were reported to also influence or co-segregate with stem cutting along with another trait i.e. heading date (1B) or plant height (3AL) (Sherman et al., 2010; Varella et al., 2015; Varella et al., 2019). Based on genomic locations of markers of minor loci detected in this study, *QSst.lrdc-1B* may be the same as the 1B locus identified previously by Sherman et al. (2010) and Varella et al. (2015). Similarly, *QSst.lrdc-2A.1* and *QSst.lrdc-2A.2* may be the same as 2A loci identified by Nilsen et al. (2017) in durum wheat, *QSst.lrdc-5A*/*QSst.lrdc-5A.1* may be same as 5A locus identified by Nilsen et al. (2017), and *QSst.lrdc-5D* may be same as 5D locus identified by Varella et al. (2015). All chromosome group 5 loci may have also been identified earlier by Larson and Macdonald (1959a), but their chromosomal locations are precisely detected in this study, particularly *QSst.lrdc-5D* which was never mapped earlier. Rest of the minor loci (*QSst.lrdc-1D*, *QSst.lrdc-2A*, *QSst.lrdc-2A.2*, *QSst.lrdc-4A.1*, *QSst.lrdc-4A.2*, *QSst.lrdc-4D*, *QSst.lrdc-5A.2*, *QSst.lrdc-5B*, *QSst.lrdc-7B*) seems to be novel and identified for the first time in this study.

#### Minor loci were largely detected in upper or middle internodes

Internode-specific QTL analysis redetected almost all whole stem solidness QTLs of the ‘Sadash/P2711’ population (except *QSst.lrdc-7B*) and ‘AAC Innova/P2711’ population (except *QSst.lrdc-1B* and *QSst.lrdc-2A.1*) (**Table 1**). Conversely, a few QTLs such as *QSst.lrdc-2D*, *QSst.lrdc-4A* and *QSst.lrdc-7B* from the ‘AAC Innova/P2711’ population were detected just for internodes but not for whole stem rating (**Table 1**). Interestingly, most minor QTLs were detected in upper or middle internodes (except *QSst.lrdc-5A.2* of ‘Sadash/P2711’ population and *QSst.lrdc-2A.2* and *QSst.lrdc-5B* of ‘AAC Innova/P2711’ population) while major QTLs were detected in all internodes rated in this study (**Table 1**). The lower stem internodes in wheat cultivars are generally filled to the greatest extent (Pluta et al., 2021) and WSS females oviposit eggs between the 2nd to 4th internode (Beres et al., 2011b). Our results indicate that although the effect of these minor QTLs on stem solidness is small and unstable, these can be useful solid stem resources to be synergistically utilized with major loci to enhance the solid stem expression in middle and upper internodes as most of the common wheat lines generally exhibit solidness in lower internodes.

### Synergistic QTL interactions enhance the expression of stem solidness

**Figure 9:**
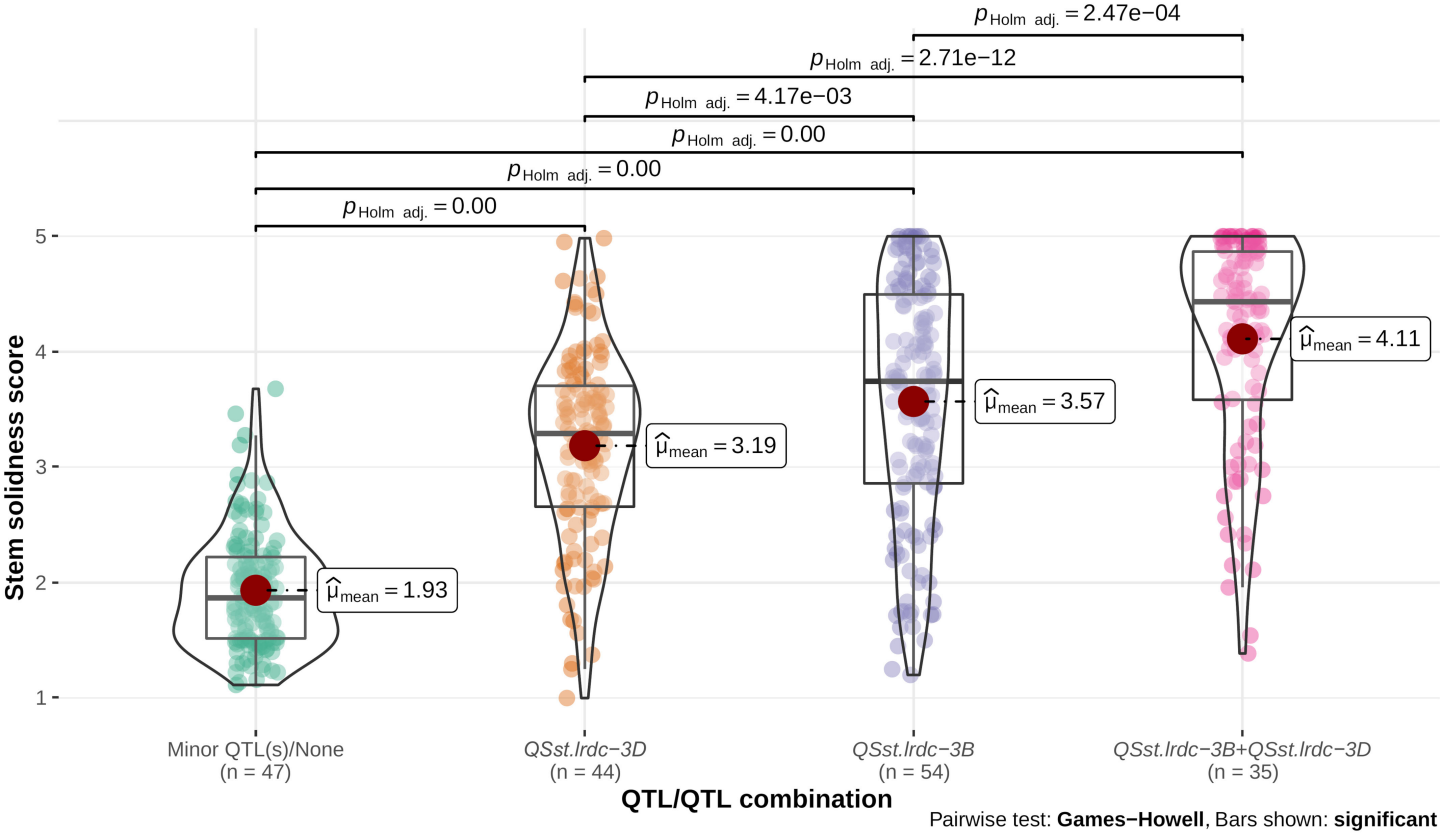
Violin plot and box plot distribution of stem solidness score in doubled haploid lines carrying (i) minor QTL(s)/none, (ii) major QTL *QSst.lrdc-3D*, (iii) major QTL *QSst.lrdc-3B*, and (iv) major QTL combination *QSst.lrdc-3B* and *QSst.lrdc-3D* in ‘Sadash/P2711’ population. The violin plots (transparent) and different color-filled circles represent the data distribution. Quartiles and medians are represented by boxes and continuous lines (gray color), respectively. The mean is shown by dark red color dots. Whiskers extend to the farthest points that are not outliers. Significant differences are shown by bar and *p* values.

**Figure 10:**
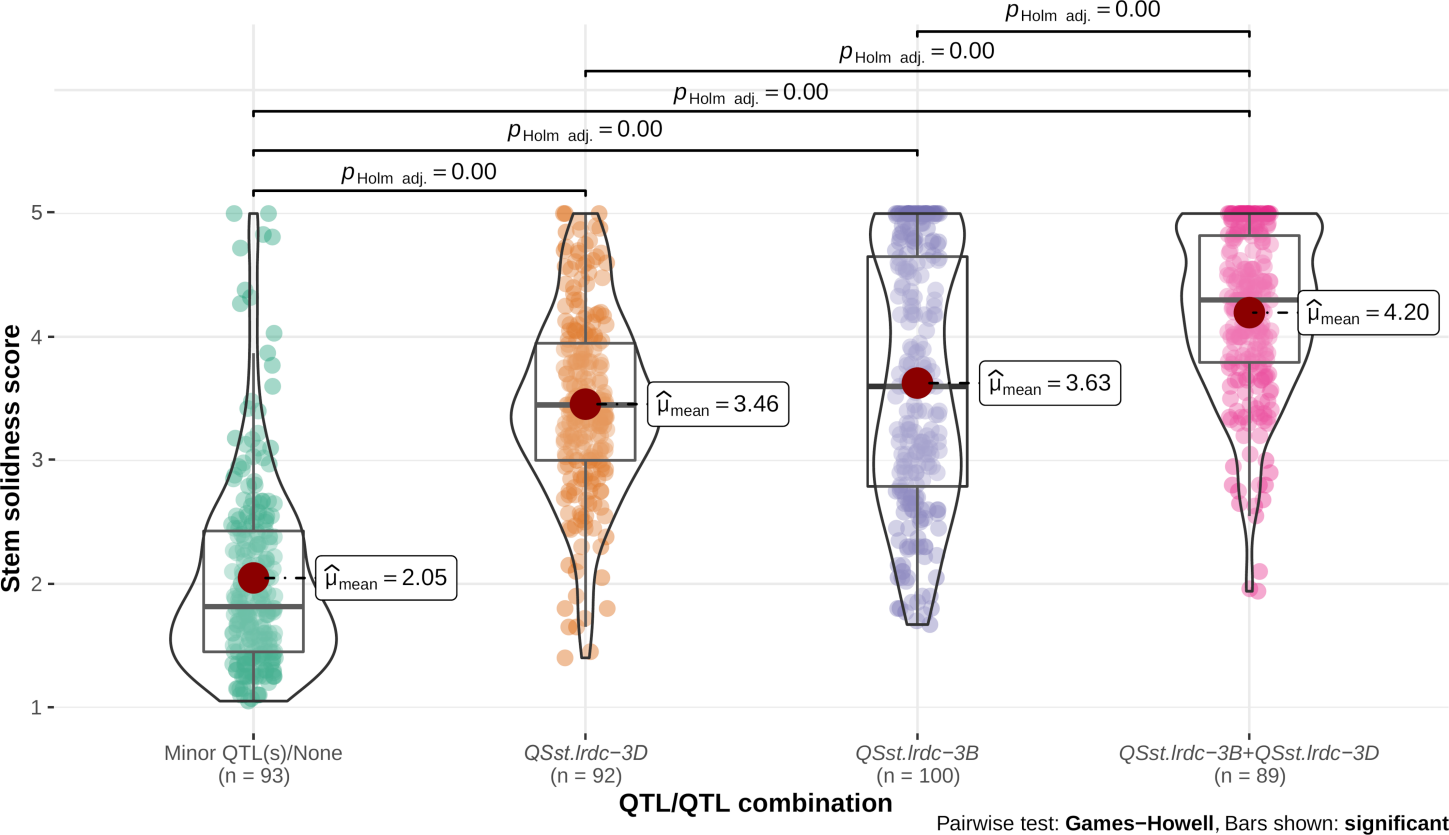
Violin plot and box plot distribution of stem solidness score in doubled haploid lines carrying (i) minor QTL(s)/none, (ii) major QTL *QSst.lrdc-3D*, (iii) major QTL *QSst.lrdc-3B*, and (iv) major QTL combination *QSst.lrdc-3B* and *QSst.lrdc-3D* in ‘AAC Innova/P2711’ population. The violin plots (transparent) and different color-filled circles represent the data distribution. Quartiles and medians are represented by boxes and continuous lines (gray color), respectively. The mean is shown by dark red color dots. Whiskers extend to the farthest points that are not outliers. Significant differences are shown by bar and *p* values.

Individual environment whole stem solidness data and genotyping data of all the DH lines for the linked markers IWB11701 and TG3069, respectively, for major QTLs, *QSst.lrdc.3B* and *QSst.lrdc-3D* of ‘Sadash/P2711’ population were analyzed to assess their effectiveness as a single QTL or QTL combination. Similarly, whole stem solidness data of all individual environments and genotyping data of the linked markers IWA1756 and IWB52401 of all the DH lines for major QTLs, *QSst.lrdc.3B* and *QSst.lrdc-3D* of ‘AAC Innova/P2711’ population were also analyzed. Solid stem data of DH lines having the same genotypic profile for each group of markers were bulked and plotted as box plot distribution. Based on the QTL profile, the recombinant DH lines in both populations were characterized into four different genotypic classes (**Figures 9**, **10**), irrespective of the solid stem alleles at minor or undetected loci. We observed that while individually, *QSst.lrdc-3B* contributed maximum stem solidness in both populations, *QSst.lrdc-3D* either conferred slightly less solidness (in the ‘Sadash/P2711’ population) or almost the same level (statistically insignificant) of solidness (in ‘AAC Innova/P2711’ population) (**Figures 9**, **10**). Thus, both major loci, *QSst.lrdc.3B* and *QSst.lrdc-3D*, were almost equally effective for pith development but together these loci produced a highly solid stem phenotype in both individual wheat populations (**Figures 9**, **10**).

Interestingly, solid stem data distribution showed that some DH lines carrying a single major gene sometimes had less solid stem than the intermediate phenotype (**Figures 9**, **10**). Conversely, some non-major gene carriers conferred stem-solidness that exceeded the intermediate phenotype (**Figures 9**, **10**). These results indicate the involvement of epistatic interactions in both populations. The phenotypic characteristics of a QTL may differ in diverse genetic backgrounds due to epistatic interaction with another gene (Dhariwal et al., 2020). This means that an apparently ‘favorable’ QTL allele at a locus may exhibit an ‘unfavorable’ or ‘unexpected’ effect or even a ‘neutral’ phenotype when introgressed to a new genetic background (Kumar et al., 2019). These interactions not only complicate the phenotypic effect but also affects the rate of genetic gain (Kumar et al., 2019), thus, an understanding of epistatic loci is vital for breeding wheat for different traits. Therefore, we further investigated the possibility of synergistic interactions between all loci. We observed that five of the identified main effect QTLs (*QSst.lrdc-2A*, *QSst.lrdc-3B*, *QSst.lrdc-3D*, *QSst.lrdc-5A.2* and *QSst.lrdc-5D*) were involved in following 5 digenic epistatic interaction: *QSst.lrdc-2A - QSst.lrdc-3B*, *QSst.lrdc-3D - QSst.lrdc-5A.2* and *QSst.lrdc-3B - QSst.lrdc-3D* in ‘Sadash/P2711’ population, and *QSst.lrdc-3B - QSst.lrdc-3D* and *QSst.lrdc-3D - QSst.lrdc-5D* in ‘AAC Innova/P2711’ population (**Supplementary Table S7**). These epistatic interactions conferred an additional increase in pith development in both populations (**Supplementary Table S7**) confirming their role in the aforementioned phenotypic differences that were observed in the same QTL profile genotypes. Particularly, the digenic epistatic interactions *QSst.lrdc-3B - QSst.lrdc-3D* and *QSst.lrdc-3D - QSst.lrdc-5D* were of high interest, which produced a similar effect as that of individual minor loci (**Table 1** and **Supplementary Table S7**). Epistatic interaction of minor loci has also been previously shown to synergistically increase the expression of 3B major locus in durum (Nilsen et al., 2017) and common wheat (Cook et al., 2004; Nilsen et al., 2017), however, this is the first report of synergistic interaction between major loci *QSst.lrdc-3B* and *QSst.lrdc-3D*. Considering digenic epistasis interactions while developing a solid stem cultivar can be useful for achieving higher pith expression in some genetic backgrounds.

## Conclusion

Our findings suggest that the genetic architecture of stem solidness in both DH populations was largely controlled by two major and stable solid stem QTLs (*QSst.lrdc-3B* and *QSst.lrdc-3D*) from solid stem line P2711 along with several minor QTLs, and some epistatic interactions among detected loci. While both major loci were detected in all internodes, minor loci were largely detected in upper and middle internodes. High expression in upper internodes can be helpful in some genetic backgrounds as solid stem genes are generally ineffective in upper internodes, particularly in common wheat cultivars. Moreover, minor loci can have relatively larger effects when deployed together or with major genes and could also be valuable for diversifying the sources of stem solidness in breeding programs. Conversely, the combination of major solid stem QTLs *QSst.lrdc-3B* and *QSst.lrdc-3D*, which produced a highly solid stem phenotype in both populations in this study, can be incorporated in modern wheat cultivars from P2711 to obtain maximum WSS resistance (Holmes, 1977). P2711 can prove as a valuable source for solid stem transfer to common wheat cultivars.

## Data availability statement

The datasets presented in this study can be found in online repositories. The names of the repository/repositories and accession number(s) can be found in the article/**Supplementary Material**.

## Author contributions

RD and HR conceived and designed the study. RD conducted all laboratory and field experiments, developed methods, performed all analyses, prepared tables and figures, interpreted the results, performed project administration and supervision, funding acquisition, wrote – the original draft and performed the review and editing. CH genotyped the mapping populations with 90K Infinium iSelect SNP assay, provided resources, and performed the review and editing. HR developed and grew the mapping populations, performed project administration and supervision, funding acquisition, provided resources, and performed the review and editing. All authors read and approved the final manuscript.

## Funding

The financial support provided by the Western Grain Research Foundation, and Alberta Wheat Commission to the AAFC Lethbridge spring wheat breeding program is gratefully acknowledged.

## Acknowledgments

The authors sincerely thank Dr. F. Koekemoer from Sensako, South Africa for providing the genetic material and Dr. Robert J. Graf from AAFC Lethbridge for providing intellectual input for conducting this study. We greatly acknowledge the technical support provided by Leslie Bihari, Julie Pepneck, Marissa Guzzi, Kim Ziegler, Kelly Ryan, Mark Virginillo (AAFC Lethbridge) in phenotyping and field experiments and Mira Popovic (AAFC Morden) for genotyping using the 90K Infinium iSelect SNP assay.

## Conflict of interest

The authors declare that the research was conducted in the absence of any commercial or financial relationships that could be construed as a potential conflict of interest.

## Publisher’s note

All claims expressed in this article are solely those of the authors and do not necessarily represent those of their affiliated organizations, or those of the publisher, the editors and the reviewers. Any product that may be evaluated in this article, or claim that may be made by its manufacturer, is not guaranteed or endorsed by the publisher.
