# Supplementary figures and images for: QTL analysis identified two major all-internodes solidness loci from a completely solid-stemmed spring wheat line
Source: Front Plant Sci. 2022 Nov 15;13:1035620. doi: 10.3389/fpls.2022.1035620 (PMC9707402; doi:10.3389/fpls.2022.1035620)

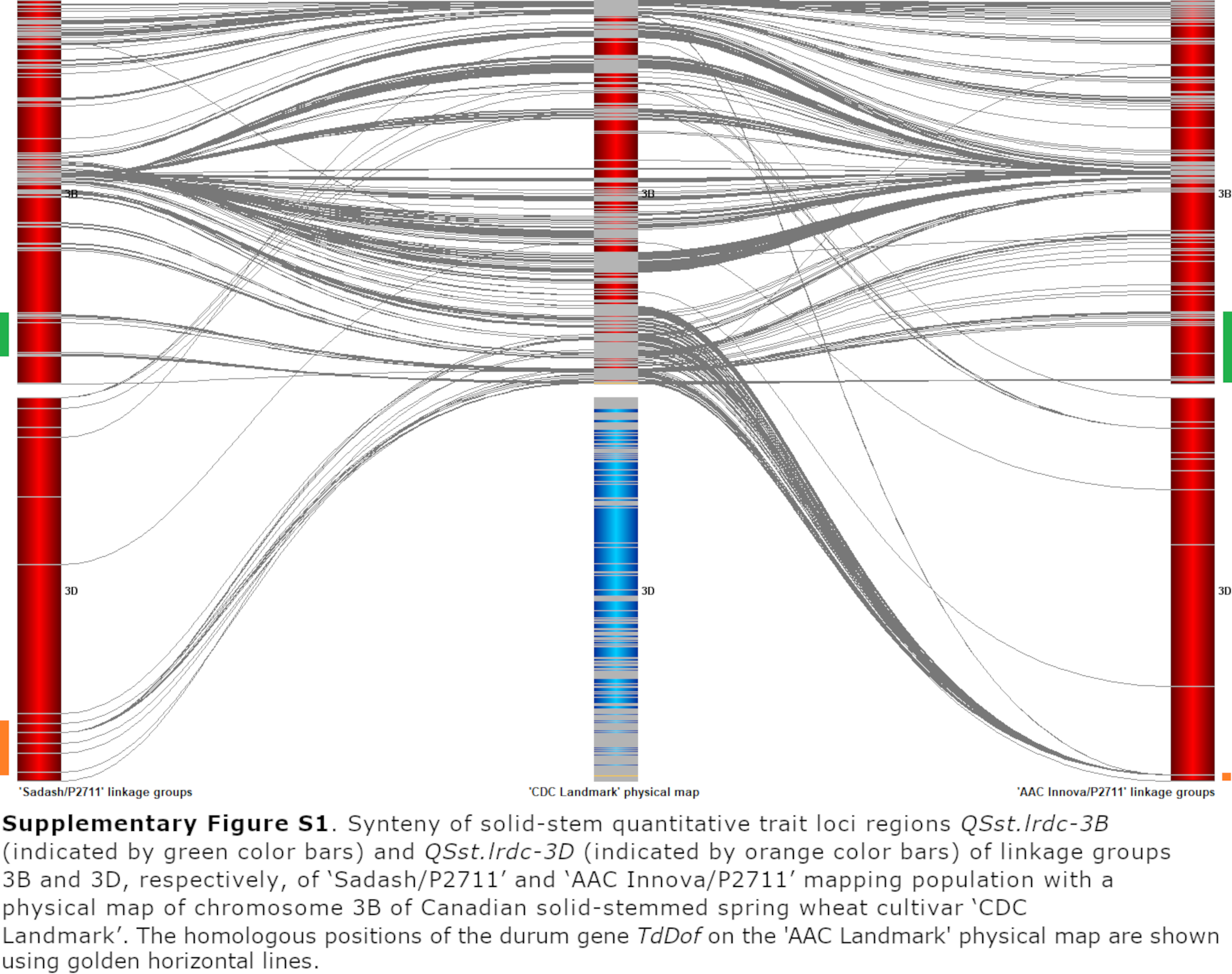

Supplement: Supplementary file 2 [file Image_1.tif]

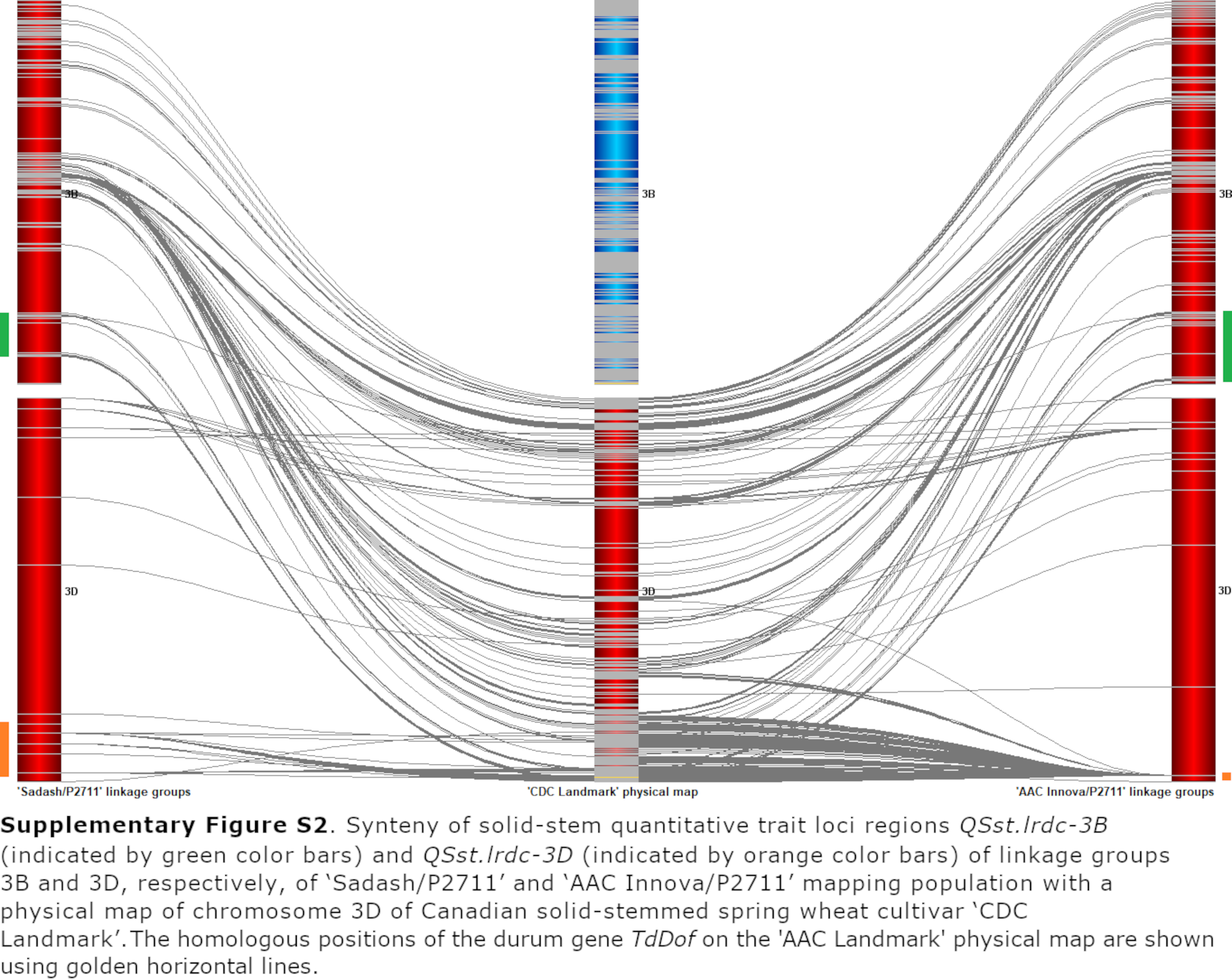

Supplement: Supplementary file 3 [file Image_2.tif]
